# Physicians’, pharmacists’, and nurses’ education of patients about antibiotic use and antimicrobial resistance in primary care settings: a qualitative systematic literature review

**DOI:** 10.3389/frabi.2024.1507868

**Published:** 2025-01-09

**Authors:** Lavinia Bianca Balea, Ragnhild J. A. Gulestø, Hongxuan Xu, Stinne Glasdam

**Affiliations:** ^1^ Private, Bucharest, Romania; ^2^ Department of Health Sciences, Institute of Nursing, VID Specialized University, Oslo, Norway; ^3^ Department of Care Sciences, Faculty of Health and Society, Malmö University, Malmö, Sweden; ^4^ Department of Health Sciences, Faculty of Medicine, Lund University, Lund, Sweden

**Keywords:** antimicrobial resistance, patient education, primary care, professionals, qualitative systematic literature review

## Abstract

**Background:**

Patients’ adherence to antibiotic treatment and related prevention of AMR is significant. Understanding healthcare professionals’ strategies for advising and educating patients in primary care settings is crucial.

**Aim:**

From the perspectives of professionals and patients, to explore how physicians, pharmacists, and nurses educate patients about antibiotic use and antimicrobial resistance in primary care settings.

**Methods:**

A qualitative systematic literature review was conducted in MEDLINE, EMBASE, CINAHL Complete, Eric, SocINDEX, PsycInfo, Web of Science and Scopus. The study included 102 publications, followed PRISMA recommendations and was registered in PROSPERO (reg.no. CRD4202455761). The studies were screened and selected based on specific inclusion and exclusion criteria using Covidence. Quality appraisal followed the Critical Appraisal Skills Program (CASP) qualitative study checklist. Data were extracted, and the analysis consisted of a descriptive numerical summary analysis and a qualitative thematic analysis.

**Results:**

The analyzed studies spanned multiple countries and settings and included perspectives of primary care physicians, pharmacists, nurses and patients. Two main themes emerged: (1) Relationships between professionals and patients influenced educational strategies, showing that trust and rapport between healthcare professionals and patients played a crucial role in shaping educational strategies around antibiotic use; (2) The organizational structures challenged professionals in guiding and educating patients, highlighting how limited resources, time constraints, and system-level pressures hindered healthcare professionals’ ability to provide consistent and effective education. Often, structural challenges led to not educating the patients on the risks of antibiotic misuse and antimicrobial resistance. The use of delayed prescriptions emerged as a strategy for improved AMR stewardship and to meet patients’ expectations for antibiotic treatment, though it raised concerns about undermining professional responsibility and authority in ensuring appropriate antibiotic use.

**Conclusion:**

Healthcare professionals’ role in educating patients about antibiotic use and AMR in primary care settings was complex, with different challenges faced by nurses, pharmacists and primary care physicians. These challenges extended beyond the clinical level, including relational, social and structural factors. Power dynamics, trust issues, and time pressures often hindered effective education on antibiotic use. Addressing gaps in education on antibiotic use and AMR requires acknowledging these multifaceted challenges, with future efforts focusing on better supporting healthcare professionals in this context.

**Systematic review registration:**

https://www.crd.york.ac.uk/prospero/, identifier CRD4202455761.

## Introduction

According to the World Health Organization (WHO, 2017), antimicrobial resistance (AMR) occurs when bacterial, viral, parasitic, and fungal microorganisms develop resistance to antimicrobial medicines. AMR is a significant contemporary social issue and a global priority for policymakers (Andersson et al., 2019; Center for Disease Dynamics, Economics & Policy, 2015; World Health Organization, 2015). In 2019, AMR was associated with 4.95 million deaths and directly attributable to 1.27 million deaths (Antimicrobial Resistance Collaborators, 2022). If unaddressed, the burden of the AMR-related disease is projected to reach 10 million deaths annually by 2050. A major driver of AMR is the misuse and overuse of antibiotics, particularly in primary care, which accounts for over 80% of antibiotic use worldwide (Wang et al., 2021). Thus, decreasing the inappropriate use of antibiotics in primary care is crucial to tackling AMR (Heyman et al., 2014).

Primary care physicians, pharmacists, and nurses are on the front lines of managing antibiotic use and advising patients on the prevention of AMR in primary care settings (Alves et al., 2021; Burnett, 2018; Sumner et al., 2018; Wong et al., 2021). Proper education regarding antibiotic use may ensure that patients understand the importance of rational antibiotic therapy, which is essential for reducing the risk of resistance (Korkmaz et al., 2024; Lambert et al., 2024; Rao et al., 2020). However, a survey-based study reveals that although 67% of patients received advice about their infection, only 8% recalled being informed about antibiotic resistance (McNulty et al., 2016). This significant gap highlights the need for improved education and communication strategies provided by healthcare professionals regarding both the proper use of antibiotics and the implications of AMR. A key prerequisite for addressing the gap is ensuring that healthcare professionals possess adequate knowledge regarding the appropriate use of antibiotics (Lalithabai et al., 2022; Lim et al., 2022; Ness et al., 2014). Research indicates that effective patient education and guidance from healthcare professionals play a crucial role in supporting antimicrobial stewardship. This involves strategies aimed at optimizing antibiotic use to prevent resistance (Ha et al., 2017; Miller et al., 2020). For instance, informing patients about the potential side effects of antibiotics and how to manage them can improve treatment compliance and health outcomes (Nieuwlaat et al., 2014). A recent review highlights that public health campaigns utilizing mass media for information dissemination, along with targeted messaging about specific infections and interactions between healthcare professionals and patients, can effectively improve public awareness of AMR and influence patients’ behavior regarding antibiotic use (Gilham et al., 2024).

Non-adherence to antibiotic treatment remains a critical challenge. Several factors influence patients’ adherence, including their knowledge of antibiotics and AMR, past experiences with infections and treatments, attitudes towards antibiotics use, as well as considerations like time and financial resources. Additionally, trust in prescribed treatment regimens and the level of social support they receive also play crucial roles in their adherence to treatment (Gualano et al., 2015; Lee et al., 2023; McCubbin et al., 2021; Pristianty et al., 2019). Responsible antibiotic use is determined not only by patient-related factors but also by a complex interplay of external influences at different levels, such as healthcare professionals’ practices, societal norms, healthcare guidelines or policies, and public health initiatives (Schmiege et al., 2020; Sievert et al., 2024). In primary care settings, physicians play a pivotal role in antibiotic prescriptions. However, they often face challenges stemming from limited knowledge or misconceptions about antibiotic use. Additionally, patient expectations and external influences, such as pharmaceutical marketing, can contribute to the overprescription of antibiotics, ultimately leading to antibiotic misuse and AMR (Md Rezal et al., 2015; Sievert et al., 2024; Sulis et al., 2020). While pharmacists and nurses are crucial in promoting antibiotic stewardship, their ability to influence prescriptions is limited once antibiotics are prescribed. This highlights the need for improved collaboration and continuous education across all healthcare professionals in primary care settings (Lim et al., 2022; Ness et al., 2014). Given that primary care’s nature and mission are to provide accessible, comprehensive, and preventive care, primary healthcare professionals are often the first point of contact for patients seeking medical care, providing them a critical position as gatekeepers to influence the appropriate use of antibiotics and reduce the occurrence of AMR (Sijbom et al., 2023; World Health Organization, 2018).

Despite these critical issues within primary care settings, existing literature primarily focuses on hospital settings or patients behaviors (Camerini et al., 2024; Giamarellou et al., 2023; Rachina et al., 2024), leaving the practices and educational strategies in primary care largely unexplored. Given the significant impact of patients’ non-adherence to antibiotic treatment and prevention of AMR, understanding the specific educational roles and strategies of healthcare professionals in primary care settings is crucial. Therefore, from the perspectives of both professionals and patients, the study aimed to explore how physicians, pharmacists, and nurses educate patients about antibiotic use and AMR in primary care settings.

## Method

This study carried out a qualitative systematic review to synthesize findings from various qualitative research studies. The method was inspired by Bettany-Saltikov and McSherry (2016). The review followed the Preferred Reporting Items for Systematic Reviews and Meta-Analyses (PRISMA) guidelines, ensuring transparency, rigor, and consistency in the systematic review process (Page et al., 2021). The review protocol is registered with PROSPERO (registration number CRD4202455761).

### Inclusion and exclusion criteria

Inclusion and exclusion criteria were established based on the PEO model (**Table 1**). The PEO model was chosen as it provided a structured approach to framing research questions and organizing data that aligns well with qualitative methodologies (Bettany-Saltikov and McSherry, 2016; Khan et al., 2004). The inclusion criteria were: 1) Primary care physicians (e.g., GPs, surgeons, and pediatricians), pharmacists, and nurses, working in primary healthcare settings or community care advising, 2) Perspectives of patients/citizens and primary care physicians, pharmacists, and nurses, 3) Qualitative studies or qualitative sub-studies in mixed method studies, 4) Published between 2014 to 2024, to reflect the most current evidence related to antibiotic stewardship, and 5) Published in English, Scandinavian or Romanian. The review excluded: 1) Systematic literature reviews, 2) Intervention studies, 3) Studies about vaccination, 4) Editorials/comments, 5) Dental care, 6) Guidelines/recommendations, and 7) Simulation studies.

**Table 1:** Populations, Exposures, and Outcomes (PEO).

Block	Population (P)	Exposure (E)	Outcome/Theme (O)
Block 1	General Practitioners, Pharmacists, Nurses, Patients, Healthcare Workers	Advising and educating patients about antibiotic use, AMR, interventions, programs, strategies	Improvement in patients’ knowledge, antibiotic use adherence, increased awareness about AMR, patient involvement
Block 2	"general practitioner*" OR physician* OR doctor* OR pharmacist* OR nurse* OR "nursing staff" OR "health professional*" OR "health care worker*" OR "healthcare worker*" OR "medical staff" OR "community health workers" OR "community nurse*" OR "community pharmacist*" OR "primary care" OR "primary healthcare" OR "community care" OR "community healthcare" OR patient* OR "health personnel"	antibiotic* OR antibiotics OR antimicrobial* OR "antibiotic use" OR "antibiotic adherence" OR AMR OR "antimicrobial resistance" OR "community pharmac*" OR "infection prevention and control measure*" OR "AMR awareness campaign*" OR educat* OR intervent* OR program* OR knowledge OR practice OR communicat* OR strateg* OR behav* OR stewardship OR "decision-making"	"improvement in knowledge" OR adherence OR "antibiotic use adherence" OR "AMR awareness" OR "patient awareness" OR “knowledge” OR "patient involvement" OR interaction* OR communication OR "decision-making" OR "infection prevention" OR stewardship OR "patient participation"

* is used as a wildcard character to represent one or more characters in a search term.

### Searching, selection, appraising, and extraction relevant data

A qualitative systematic literature review was conducted in MEDLINE, EMBASE, CINAHL Complete, Eric, SocINDEX, PsycInfo, and Web of Science (Last search 8 July 2024), supported by an experienced librarian. The initial search retrieved 9948 publications, which were transferred to Covidence software for screening. The search strategies are presented in **Table 2**. To identify additional relevant studies, a citation pearl search was conducted in the Scopus database (Last search 1 August 2024).

**Table 2:** The full electronic search strategy for all three databases.

Database	Search String
PubMed (National Library of Medicine) Limiters >Language: Danish, English, Norwegian, Romanian, Swedish Published Date: 2014 - 2024 Date of search: 2024/06/12 Hits: 4029 records	#1 "general practitioner*"[Title/Abstract] OR physician*[Title/Abstract] OR doctor*[Title/Abstract] OR pharmacist*[Title/Abstract] OR nurse*[Title/Abstract] OR "nursing staff"[Title/Abstract] OR "health professional*"[Title/Abstract] =1033951 records #2 (antibiotic*[Title/Abstract] OR antibiotics[Title/Abstract] OR antimicrobial[Title/Abstract] OR "community pharmac*"[Title/Abstract]) AND (educat*[Title/Abstract] OR intervent*[Title/Abstract] OR program*[Title/Abstract] OR knowledge[Title/Abstract] OR practice[Title/Abstract] OR communicat*[Title/Abstract] OR strateg*[Title/Abstract] OR behav*[Title/Abstract] OR stewardship[Title/Abstract] OR “decision-making”) =140421 records #3 "infection prevention and control measure*" OR "AMR awareness campaign*" =512 records #4 #2 OR #3 =140857 records #5 #1 AND #4 =17601 records #6 primary care[Title/Abstract] OR primary healthcare[Title/Abstract] OR community care[Title/Abstract] OR community healthcare[Title/Abstract] OR community nurse*[Title/Abstract] OR community pharmacist*[Title/Abstract] =174252 records #7 "Primary Health Care"[Mesh] =197855 records #8 #6 OR #7 =306271 records #9 #5 AND #8 =5856 records #8 #7 Filters: Danish, English, Norwegian, Romanian, Swedish, from 2014 - 2024 =**4029 records**
Embase.com (Elsevier, 1947-present) Date of search: 2024/06/12 Hits: 1538 records	#1 'general practitioner*':ti,ab,kw OR physician*:ti,ab,kw OR doctor*:ti,ab,kw OR pharmacist*:ti,ab,kw OR nurse*:ti,ab,kw OR 'nursing staff':ti,ab,kw OR 'health professional*':ti,ab,kw =1436757 records #2 (antibiotic*:ti,ab,kw OR antibiotics:ti,ab,kw OR antimicrobial:ti,ab,kw OR 'community pharmac*':ti,ab,kw) AND (educat*:ti,ab,kw OR intervent*:ti,ab,kw OR program*:ti,ab,kw OR knowledge:ti,ab,kw OR practice:ti,ab,kw OR communicat*:ti,ab,kw OR strateg*:ti,ab,kw OR behav*:ti,ab,kw OR stewardship:ti,ab,kw OR 'decision-making':ti,ab,kw) =198528 records #3 'infection prevention and control measure*' OR 'amr awareness campaign*' =598 records #4 #2 OR #3 =199030 records #5 #1 AND #4 =31163 records #6 'primary health care'/exp OR 'primary care':ti,ab,kw OR 'primary healthcare':ti,ab,kw OR 'community care':ti,ab,kw OR 'community healthcare':ti,ab,kw OR 'community nurse*':ti,ab,kw OR 'community pharmacist*':ti,ab,kw =314354 records #7 #5 AND #6 =9730 records #8 #7 AND (2014:py OR 2015:py OR 2016:py OR 2017:py OR 2018:py OR 2019:py OR 2020:py OR 2021:py OR 2022:py OR 2023:py OR 2024:py) AND [embase]/lim NOT ([embase]/lim AND [medline]/lim) =3729 records #9 #7 AND (2014:py OR 2015:py OR 2016:py OR 2017:py OR 2018:py OR 2019:py OR 2020:py OR 2021:py OR 2022:py OR 2023:py OR 2024:py) AND [embase]/lim NOT ([embase]/lim AND [medline]/lim) AND ([danish]/lim OR [english]/lim OR [norwegian]/lim OR [romanian]/lim OR [swedish]/lim) =3627 records #10 #9 AND 'conference abstract'/it =2089 records #11 #9 NOT #10 =1538 records
CINAHLComplete (Cumulative Index to Nursing and Allied Health Literature; EbscoHost, inception to present) Date of search: 2024/06/13 Limiters Publication Date: 2014/01/01-2024/12/31 Language: English Hits: 2996 records	#1 TI ( "general practitioner*" OR physician* OR doctor* OR pharmacist* OR nurse* OR "nursing staff" OR "health professional*" ) OR AB ( "general practitioner*" OR physician* OR doctor* OR pharmacist* OR nurse* OR "nursing staff" OR "health professional*" ) =633047 records #2 TI ( antibiotic* OR antibiotics OR antimicrobial OR "community pharmac*" ) OR AB ( antibiotic* OR antibiotics OR antimicrobial OR "community pharmac*" ) =83901 records #3 TI ( educat* OR intervent* OR program* OR knowledge OR practice OR communicat* OR strateg* OR behav* OR stewardship OR “decision-making”) ) OR AB ( educat* OR intervent* OR program* OR knowledge OR practice OR communicat* OR strateg* OR behav* OR stewardship OR “decision-making” ) =2134431 #4 #2 AND #3 =27467 records #5 “infection prevention and control measure*" OR "AMR awareness campaign*" =190 records #6 (MH "Prescriptions, Drug+") AND TI ( antibiotic OR antibiotics ) =709 records #7 #4 OR #5 OR #6 =27937 records #8 #1 AND #7 =7202 records #9 (MM "Primary Health Care") OR TI ( primary care OR primary healthcare OR community care OR community healthcare OR community nurse* OR community pharmacist* ) OR AB ( primary care OR primary healthcare OR community care OR community healthcare OR community nurse* OR community pharmacist* ) =271695 records #10 #8 AND #9 =3981 records #11 #10 Limiters - Publication Date: 20140101-20241231 Narrow by Language: - English =2996 records
PsycInfo (EbscoHost, inception to present) Date of search: 2024/06/13 Limiters Publication Year: 2014-2024 Language: English Hits: 593 records	#1 TI ( "general practitioner*" OR physician* OR doctor* OR pharmacist* OR nurse* OR "nursing staff" OR "health professional*" ) OR AB ( "general practitioner*" OR physician* OR doctor* OR pharmacist* OR nurse* OR "nursing staff" OR "health professional*" ) =224732 records #2 TI ( (antibiotic* OR antibiotics OR antimicrobial OR "community pharmac*" ) OR AB ( (antibiotic* OR antibiotics OR antimicrobial OR "community pharmac*" ) =5225 records #3 TI ( educat* OR intervent* OR program* OR knowledge OR practice OR communicat* OR strateg* OR behav* OR stewardship OR “decision-making” ) OR AB ( educat* OR intervent* OR program* OR knowledge OR practice OR communicat* OR strateg* OR behav* OR stewardship OR “decision-making” ) =2808907 records #4 #2 AND #3 =2995 records #5 “infection prevention and control measure*" OR "AMR awareness campaign*" =14 records #6 #4 OR #5 =3008 records #7 #1 AND #6 =1286 records #8 MM "Primary Health Care" OR TI ( primary care OR primary healthcare OR community care OR community healthcare OR community nurse* OR community pharmacist* ) OR AB ( primary care OR primary healthcare OR community care OR community healthcare OR community nurse* OR community pharmacist* ) =127385 records #8 #7 AND #8 =934 records #9 #8 Limiters - Publication Year: 2014-2024 Narrow by Language: - English =593 records
ERIC (Ebsco Host] Date of search: 2024/06/13 Limiters Published Date: 2014/01/01-2022/12/31 Hits: 6 records	#1 TI ( "general practitioner*" OR physician* OR doctor* OR pharmacist* OR nurse* OR "nursing staff" OR "health professional*" ) OR AB ( "general practitioner*" OR physician* OR doctor* OR pharmacist* OR nurse* OR "nursing staff" OR "health professional*" ) =35777 records #2 TI ( antibiotic* OR antibiotics OR antimicrobial OR "community pharmac*" ) OR AB ( antibiotic* OR antibiotics OR antimicrobial OR "community pharmac*" ) =292 records #3 TI ( educat* OR intervent* OR program* OR knowledge OR practice OR communicat* OR strateg* OR behav* OR stewardship OR “decision-making” ) OR AB ( educat* OR intervent* OR program* OR knowledge OR practice OR communicat* OR strateg* OR behav* OR stewardship OR “decision-making” ) =1362491 records #4 #2 AND #3 =171 records #5 TI ( “infection prevention and control measure*" OR "AMR awareness campaign*" ) OR AB ( “infection prevention and control measure*" OR "AMR awareness campaign*" ) =1 records #6 #4 OR #5 =172 records #7 #1 AND #6 =49 records #8 TI ( primary care OR primary healthcare OR community care OR community healthcare OR community nurse* OR community pharmacist* ) OR AB ( primary care OR primary healthcare OR community care OR community healthcare OR community nurse* OR community pharmacist* ) =16472 records #9 #7 AND #8 =35 records #10 #9 Limiters - Published Date: 20140101-20221231 =6 records
SocINDEX with Full Text (EBSCOhost) Date of search: 2024/06/13 Limiters Publication Date: 2014/01/01-2024/12/31 Hits: 49 records	#1 TI ( "general practitioner*" OR physician* OR doctor* OR pharmacist* OR nurse* OR "nursing staff" OR "health professional*" ) OR AB ( "general practitioner*" OR physician* OR doctor* OR pharmacist* OR nurse* OR "nursing staff" OR "health professional*" ) =71527 records #2 TI ( antibiotic* OR antibiotics OR antimicrobial OR "community pharmac*" ) OR AB ( antibiotic* OR antibiotics OR antimicrobial OR "community pharmac*" ) =3044 records #3 TI ( educat* OR intervent* OR program* OR knowledge OR practice OR communicat* OR strateg* OR behav* OR stewardship OR “decision-making” ) OR AB ( educat* OR intervent* OR program* OR knowledge OR practice OR communicat* OR strateg* OR behav* OR stewardship OR “decision-making” ) =1074444 records #4 #2 OR #3 =1027 records #5 “infection prevention and control measure*" OR "AMR awareness campaign*" =4 records #6 #4 OR #5 =1031 records #7 #1 AND #6 =410 records #8 TI ( primary care OR primary healthcare OR community care OR community healthcare OR community nurse* OR community pharmacist* ) OR AB ( primary care OR primary healthcare OR community care OR community healthcare OR community nurse* OR community pharmacist* ) =39152 records #9 #7 AND #8 =226 records #10 #9 Limiters - Publication Date: 20140101-20241231 =49 records
Web of Science Core collection (Clarivate Analytics) Date of search:2024/06/14 Limiters Publication Years: 2014 or 2015 or 2016 or 2017 or 2018 or 2019 or 2020 or 2021 or 2022 or 2023 or 2024; Document Types: Article or Review Article Languages: English Hits: 6753 records	#1 AB=(("general practitioner*" OR physician* OR doctor* OR pharmacist* OR nurse* OR "nursing staff" OR "health professional*")) =721288 records #2 TI=("general practitioner*" OR physician* OR doctor* OR pharmacist* OR nurse* OR "nursing staff" OR "health professional*") =291411 records #3 #1 OR #2 =881268 records #4 (TS=(antibiotic* OR antibiotics OR antimicrobial OR "community pharmac*")) AND TS=(educat* OR intervent* OR program* OR knowledge OR practice OR communicat* OR strateg* OR behav* OR stewardship OR “decision-making”) =174354 records #5 ALL=(“infection prevention and control measure*" OR "AMR awareness campaign*") =472 records #6 #4 OR #5 =174745 records #7 #3 AND #6 =17831 records #8 TS=(primary care OR primary healthcare OR community care OR community healthcare OR community nurse* OR community pharmacist*) =579837 records #9 #7 AND #8 =9985 records #10 #9 and 2014 or 2015 or 2016 or 2017 or 2018 or 2019 or 2020 or 2021 or 2022 or 2023 or 2024 (Publication Years) and Article or Review Article (Document Types) and English (Languages) =6753 records

**Figure 1:**
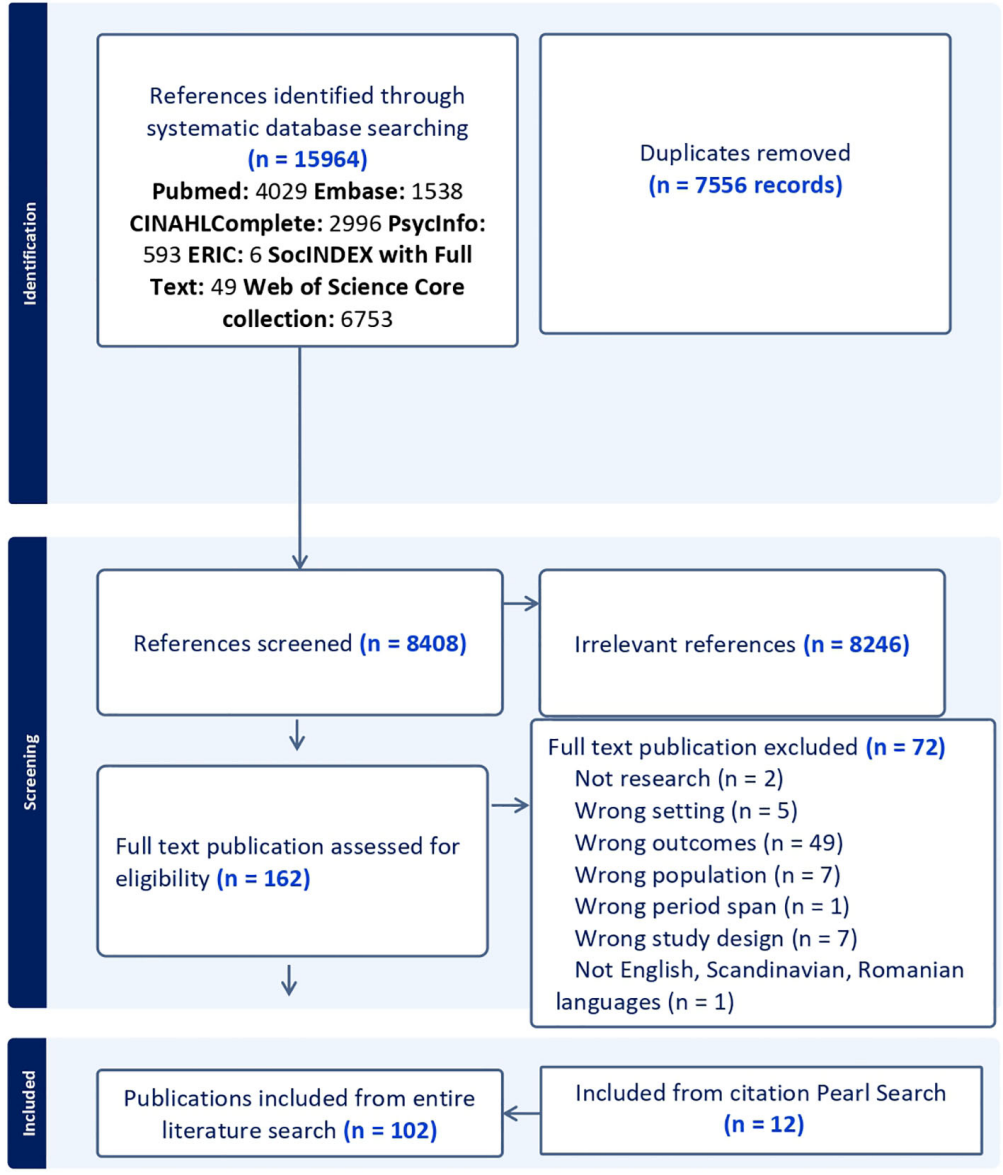
PRISMA 2020 flow diagram of study selection here.

Two of the authors (LBB and SG) collaborated on the study selection process. In cases of disagreement during the screening, full-text review, or citation search processes, discussions were held with the other authors (RJAG and HX) until a consensus was reached. A PRISMA flow diagram illustrates the study selection process (**Figure 1**). As a second opinion, RJAG reviewed all the included articles in relation to the inclusion and exclusion criteria, which supported the selection. The 102 included publications are marked with an asterisk (*) in the reference list.

The quality of the included publications was assessed using the Critical Appraisal Skills Program (CASP) qualitative study checklist (Critical Appraisal Skills Program, 2018). The use of the CASP qualitative study checklist provided a systematic method for assessing the quality of included publications, demonstrating a commitment to methodological rigor and ensuring the review’s findings were grounded in credible, high-quality evidence. The use of the checklist was endorsed by the Cochrane Qualitative and Implementation Methods Group (Long et al., 2020). This checklist consists of ten questions evaluating various aspects of the studies, including their aim, methodology and design, recruitment strategy, data collection, data analysis, findings, and overall research value (see **Table 3**). The purpose of the quality appraisal was to ensure the strength of the evidence in addressing our research question.

**Table 3:** Qualitative study appraisal*.

	Section A: Are the results valid?						Section B: What are the results?			Section C: Will the results help locally?	Scores
Author(s), years	1. Was there a clear statement of the aims of the research?	2. Is a qualitative methodology appropriate?	3. Was the research design appropriate to address the aims of the research?	4. Was the recruitment strategy appropriate to the aims of the research?	5. Was the data collected in a way that addressed the research issue?	6. Has the relationship between researcher and participants been adequately considered?	7. Have ethical issues been taken into consideration?	8. Was the data analysis sufficiently rigorous?	9. Is there a clear statement of findings?	10. How valuable is the research?	
Alhomoud et al., 2018	Yes	Yes	Yes	Yes	Yes	Yes	Yes	Yes	Yes	Yes	10/10
Alkadhimi et al., 2020	Yes	Yes	Yes	Yes	Yes	Cannot tell	Yes	Yes	Yes	Yes	9/10
Alkirawan et al., 2022	Yes	Yes	Yes	Yes	Yes	Yes	Yes	Yes	Yes	Yes	10/10
Alzard et al., 2024	Yes	Yes	Yes	Yes	Yes	Cannot tel	Yes	Yes	Yes	Yes	9/10
Amin et al., 2017	Yes	Yes	Yes	Yes	Yes	Yes	Yes	Yes	Yes	Yes	10/10
Anderson et al., 2019	Yes	Yes	Yes	Yes	Yes	Cannot tell	Yes	Yes	Yes	Yes	9/10
Aponte-González et al., 2019	Yes	Yes	Yes	Yes	Yes	Yes	Yes	Yes	Yes	Yes	10/10
Arnau-Sánchez et al., 2023	Yes	Yes	Yes	Yes	Yes	Yes	Yes	Yes	Yes	Yes	10/10
Ashdown et al., 2021	Yes	Yes	Yes	Yes	Yes	Yes	Yes	Yes	Yes	Yes	10/10
Atif et al., 2020	Yes	Yes	Yes	Yes	Yes	Cannot tell	Yes	Yes	Yes	Yes	9/10
Bergsholm et al., 2023a	Yes	Yes	Yes	Yes	Yes	Yes	Yes	Yes	Yes	Yes	10/10
Bergsholm et al., 2023b	Yes	Yes	Yes	Yes	Yes	Yes	Yes	Yes	Yes	Yes	10/10
Biezen et al., 2017	Yes	Yes	Yes	Yes	Yes	Cannot tell	Yes	Yes	Yes	Yes	9/10
Biezen et al., 2019	Yes	Yes	Yes	Yes	Yes	Cannot tell	Yes	Yes	Yes	Yes	9/10
Bisgaard et al., 2021	Yes	Yes	Yes	Yes	Yes	Cannot tell	Yes	Yes	Yes	Yes	9/10
Black et al., 2014	Yes	Yes	Yes	Yes	Yes	Yes	Yes	Yes	Yes	Yes	10/10
Bless et al., 2016	Yes	Yes	Yes	Yes	Yes	Cannot tell	Yes	Yes	Yes	Yes	9/10
Boaitey et al., 2023	Yes	Yes	Yes	Yes	Yes	Cannot tell	Yes	Yes	Yes	Yes	9/10
Boiko et al., 2020	Yes	Yes	Yes	Yes	Yes	Yes	Yes	Yes	Yes	Yes	10/10
Bordado Sköld, et al, 2017	Yes	Yes	Yes	Yes	Yes	Yes	Yes	Yes	Yes	Yes	10/10
Bosley et al., 2021	Yes	Yes	Cannot tell	Yes	Yes	Cannot tell	Yes	Yes	Yes	Yes	8/10
Brisley et al., 2023	Yes	Yes	Yes	Yes	Yes	Cannot tell	Yes	Cannot tell	Yes	Yes	8/10
Brookes-Howell et al., 2014	Yes	Yes	Yes	Yes	Yes	Cannot tell	Yes	Yes	Yes	Yes	9/10
Cabral, C., Ingram, J., Hay, A.D. & Horwood, J., 2014	Yes	Yes	Yes	Yes	Yes	Cannot tell	Yes	Yes	Yes	Yes	9/10
Cabral et al., 2016	Yes	Yes	Yes	Yes	Yes	Cannot tell	Yes	Yes	Yes	Yes	9/10
Colliers et al., 2018	Yes	Yes	Yes	Yes	Yes	Yes	Yes	Yes	Yes	Yes	10/10
Colliers et al., 2020	Yes	Yes	Yes	Yes	Yes	Yes	Yes	Yes	Yes	Yes	10/10
Courtenay et al., 2017	Yes	Yes	Yes	Yes	Yes	Cannot tell	Yes	Yes	Yes	Yes	9/10
Courtenay et al., 2019	Yes	Yes	Yes	Yes	Yes	Cannot tell	Yes	Yes	Yes	Yes	9/10
Cox et al., 2023	Yes	Yes	Yes	Yes	Yes	Cannot tell	Yes	Yes	Yes	Yes	9/10
Dallas et al., 2020	Yes	Yes	Yes	Yes	Yes	Yes	Yes	Yes	Yes	Yes	10/10
Darj et al., 2019	Yes	Yes	Yes	Yes	Yes	Cannot tell	Yes	Yes	Yes	Yes	9/10
Dempsey et al., 2014											
Duane et al., 2016 (Ireland)	Yes	Yes	Yes	Yes	Yes	Cannot tell	Yes	Yes	Yes	Yes	9/10
Durand et al., 2022 (France)	Yes	Yes	Yes	Yes	Yes	Cannot tell	Yes	Yes	Yes	Yes	9/10
Essilini et al., 2021 (France)	Yes	Yes	Yes	Yes	Yes	Cannot tell	Yes	Yes	Yes	Yes	9/10
Fletcher-Lartey et al., 2016 (Australia)	Yes	Yes	Yes	Yes	Yes	Cannot tell	Yes	Yes	Yes	Yes	9/10
Gaarslev et al., 2016 (Australia)	Yes	Yes	Yes	Yes	Yes	Cannot tell	Yes	Yes	Yes	Yes	9/10
Gautham et al., 2024 (India)	Yes	Yes	Yes	Yes	Yes	Yes	Yes	Yes	Yes	Yes	10/10
Ghiga and Stålsby Lundborg, 2016 (Romania)	Yes	Yes	Yes	Cannot tell	Yes	Yes	Yes	Yes	Yes	Yes	9/10
Ghiga et al., 2023 (Romania)	Yes	Yes	Yes	No	Yes	Cannot tell	Yes	Cannot tell	Yes	Yes	8/10
Grigoryan et al., 2022 (United States and Germany)	Yes	Yes	Yes	Yes	Yes	Yes	Yes	Yes	Yes	Yes	10/10
Guo et al., 2021 (Singapore)	Yes	Yes	Yes	Yes	Yes	Yes	Yes	Yes	Yes	Yes	10/10
Halls et al., 2017 (United Kingdom)	Yes	Yes	Yes	No	Yes	Cannot tell	Yes	Yes	Yes	Yes	9/10
Hika et al., 2022 (New Zealand)	Yes	Yes	Yes	Yes	Yes	Cannot tell	Yes	Yes	Yes	Yes	9/10
Hoang et al., 2024 (Vietnam)	Yes	Yes	Yes	No	Yes	Cannot tell	Yes	Yes	Yes	Yes	9/10
Horwood et al., 2016 (United Kingdom)	Yes	Yes	Yes	No	Yes	Cannot tell	Yes	Yes	Yes	Yes	9/10
Hu et al., 2024 (United Kingdom)	Yes	Yes	Yes	Yes	Yes	Cannot tell	Yes	Yes	Yes	Yes	9/10
Jakupi et al., 2019 (Kosovo)	Yes	Yes	Yes	Yes	Yes	Cannot tell	Yes	Yes	Yes	Yes	9/10
Jones et al., 2018 (United Kingdom)	Yes	Yes	Yes	Yes	Yes	Cannot tell	Yes	Yes	Yes	Yes	9/10
Kaminsky et al., 2020 (Sweden)	Yes	Yes	Yes	Yes	Yes	Cannot tell	Yes	Yes	Yes	Yes	9/10
Khan et al., 2021 (Pakistan)	Yes	Yes	Yes	Yes	Yes	Cannot tell	Yes	Yes	Yes	Yes	9/10
Khan et al., 2022 (Pakistan)	Yes	Yes	Yes	Yes	Yes	Cannot tell	Yes	Yes	Yes	Yes	9/10
Knobloch et al., 2021 (United States of America)	Yes	Yes	Yes	Yes	Yes	Cannot tell	Yes	Yes	Yes	Yes	9/10
Kurotschka et al., 2024 (Germany)	Yes	Yes	Yes	Yes	Yes	Cannot tell	Yes	Yes	Yes	Yes	9/10
Laytner et al., 2023 (United States of America)	Yes	Yes	Yes	Yes	Yes	Cannot tell	Yes	Yes	Yes	Yes	9/10
Lescure et al., 2022 (The Netherlands)	Yes	Yes	Yes	Yes	Yes	Yes	Yes	Yes	Yes	Yes	10/10
Lipstein et al., 2019 (United States of America)	Yes	Yes	Yes	Yes	Yes	Yes	Yes	Yes	Yes	Yes	10/10
Lum et al., 2017 (Australia)	Yes	Yes	Yes	Yes	Yes	Yes	Yes	Yes	Yes	Yes	10/10
Lum et al., 2018 (Australia)	Yes	Yes	Yes	Yes	Yes	Cannot tell	Yes	Yes	Yes	Yes	9/10
Mahmoud et al., 2018 (Saudi Arabia)	Yes	Yes	Yes	Yes	Yes	Cannot tell	Yes	Yes	Yes	Yes	9/10
Manderson, L., 2020 (South Africa)	Yes	Yes	Yes	Yes	Yes	Cannot tell	Yes	Yes	Yes	Yes	9/10
Mas-Dalmau et al., 2023 (Spain)	Yes	Yes	Yes	Yes	Yes	Cannot tell	Yes	Yes	Yes	Yes	9/10
McDermott, L., Leydon, G. M., Halls, A., Kelly, J., Nagle, A., White, J. & Little, P., 2017 (United Kingdom)	Yes	Yes	Yes	Yes	Yes	Cannot tell	Yes	Yes	Yes	Yes	9/10
Medina-Perucha et al., 2020 (Spain)	Yes	Yes	Yes	Yes	Yes	Cannot tell	Yes	Yes	Yes	Yes	9/10
Mortazhejri et al., 2020 (Canada)	Yes	Yes	Yes	Yes	Yes	Yes	Yes	Yes	Yes	Yes	10/10
Musoke et al., 2023 (Uganda)	Yes	Yes	Yes	Yes	Yes	Yes	Yes	Yes	Yes	Yes	10/10
Mustafa et al., 2014 (United Kingdoms)	Yes	Yes	Yes	Yes	Yes	Cannot tell	Yes	Yes	Yes	Yes	9/10
O'Doherty et al., 2019 (Ireland)	Yes	Yes	Yes	Yes	Yes	Yes	Yes	Yes	Yes	Yes	10/10
Om et al., 2017 (Cambodia)	Yes	Yes	Yes	Yes	Yes	Cannot tell	Yes	Yes	Yes	Yes	9/10
Özcebe et al., 2022 (Turkey, Germany, Sweden and the Netherlands)	Yes	Yes	Yes	Yes	Yes	Yes	Yes	Yes	Yes	Yes	10/10
Peiffer-Smadja et al., 2020 (United Kingdom)	Yes	Yes	Yes	Yes	Yes	Cannot tell	Yes	Yes	Yes	Yes	9/10
Poss-Doering et al., 2020 (Germany)	Yes	Yes	Yes	Yes	Yes	Cannot tell	Yes	Yes	Yes	Yes	9/10
Raspopovic et al., 2016 (Montenegro)	Yes	Yes	Yes	Yes	Yes	Cannot tell	Yes	Yes	Yes	Yes	9/10
Res et al., 2017 (Australia)	Yes	Yes	Yes	Yes	Yes	Cannot tell	Yes	Yes	Yes	Yes	9/10
Ryves et al., 2016 (United Kingdom)	Yes	Yes	Yes	Yes	Yes	Cannot tell	Yes	Yes	Yes	Yes	9/10
Saleem et al., 2019 (Pakistan)	Yes	Yes	Yes	Yes	Yes	Cannot tell	Yes	Yes	Yes	Yes	9/10
Saliba-Gustafsson et al., 2021 (Malta)	Yes	Yes	Yes	Yes	Yes	Cannot tell	Yes	Yes	Yes	Yes	9/10
Saliba-Gustafsson et al., 2019 (Malta)	Yes	Yes	Yes	Yes	Yes	Cannot tell	Yes	Yes	Yes	Yes	9/10
Salim and Elgizoli, 2017 (Sudan)											
Sargent et al., 2017 (Australia)	Yes	Yes	Yes	Yes	Yes	Yes	Yes	Yes	Yes	Yes	10/10
Sayood et al., 2021 (United States of America)	Yes	Yes	Yes	Yes	Yes	Cannot tell	Yes	Yes	Yes	Yes	9/10
Sharaf et al., 2021 (Qatar)	Yes	Yes	Yes	Yes	Yes	Yes	Yes	Yes	Yes	Yes	10/10
Simeoni et al., 2022 (Canada)	Yes	Yes	Yes	Yes	Yes	Yes	Yes	Yes	Yes	Yes	10/10
Souto-López et al., 2020 (Spain)	Yes	Yes	Yes	Yes	Yes	Yes	Yes	Yes	Yes	Yes	10/10
Spicer et al., 2020, (United States of America)	Yes	Yes	Yes	Yes	Yes	Cannot tell	Yes	Yes	Yes	Yes	9/10
Stivers and Timmermans, 2021 (United States of America)	Yes	Yes	Yes	Yes	Yes	Cannot tell	Yes	Yes	Yes	Yes	9/10
Sundvall et al., 2020 (Sweden)	Yes	Yes	Yes	Yes	Yes	Yes	Yes	Yes	Yes	Yes	10/10
Suy et al., 2019 (Cambodia)	Yes	Yes	Yes	Yes	Yes	Yes	Yes	Yes	Yes	Yes	10/10
Sychareun et al., 2022 (Lao PDR)	Yes	Yes	Yes	Yes	Yes	Cannot tell	Yes	Yes	Yes	Yes	9/10
Thaggard et al., 2023 (New Zealand)	Yes	Yes	Yes	Yes	Yes	Yes	Yes	Yes	Yes	Yes	10/10
Tonna et al., 2020 (United Kingdom)	Yes	Yes	Yes	Yes	Yes	Cannot tell	Yes	Yes	Yes	Yes	9/10
Torres et al., 2020 (Mozambique)	Yes	Yes	Yes	Yes	Yes	Cannot tell	Yes	Yes	Yes	Yes	9/10
Torres et al, 2023 (Mozambique)	Yes	Yes	Yes	Yes	Yes	Cannot tell	Yes	Yes	Yes	Yes	9/10
van der Zande et al., 2019 (United Kingdom)	Yes	Yes	Yes	Yes	Yes	Cannot tell	Yes	Yes	Yes	Yes	9/10
van Hecke et al., 2019a (United Kingdom)	Yes	Yes	Yes	Yes	Yes	Yes	Yes	Yes	Yes	Yes	10/10
van Hecke et al., 2019b (South Africa)	Yes	Yes	Yes	Yes	Yes	Yes	Yes	Yes	Yes	Yes	10/10
van Horrik et al., 2024 (The Netherlands)	Yes	Yes	Yes	Yes	Yes	Cannot tell	Yes	Yes	Yes	Yes	9/10
Williams et al., 2018 (United Kingdom)	Yes	Yes	Yes	Yes	Yes	Cannot tell	Yes	Yes	Yes	Yes	9/10
Yates et al., 2018 (United States of America)	Yes	Yes	Yes	Yes	Yes	Cannot tell	Yes	Yes	Yes	Yes	9/10
Zago et al., 2023 (Brazil)	Yes	Yes	Yes	Yes	Yes	Cannot tell	Yes	Yes	Yes	Yes	9/10
Zetts et al., 2020 (United States of America)	Yes	Yes	Yes	Yes	Yes	Cannot tell	Yes	Yes	Yes	Yes	9/10

Conducted in accordance with CASP Qualitative study checklist (Critical Appraisal Skills Programme, 2018).

### Strategy for data analysis

The data analysis strategy included a descriptive numerical summary analysis, presented as ‘Characteristics of the Studies’, and an inductive thematic analysis inspired by Braun and Clarke (2006).

First, the publications were read multiple times, facilitating familiarization with the material (Braun and Clarke, 2006). The following data were extracted: 1) Authors, 2) Location, 3) Journal, 4) Study period, 5) Study design, 6) Sample size, 7) Target group and context, 8) Theory/concepts, 9) Results, and 10) Limitations. Data extraction focused on the qualitative findings relevant to the review aim (Bettany-Saltikov and McSherry, 2016). The included studies represent diverse contexts and countries, each with unique cultural and healthcare system characteristics. To manage this heterogeneity, we focused on extracting data that was applicable across various settings while noting contextual differences. All authors checked the extracted data for accuracy. A selection of this data extraction is presented in **Table 4**.

**Table 4:** Study characteristics.

Author(s), Year of Publication (Country)	Journal (Year: Impact Factor)	Study Aim	Design; Study Population; Setting	Study Period
Alhomoud et al., 2018 (Saudi Arabia)	BMC Health Service Research (2023: 7,0)	To explore reasons for non-prescribed sale of antibiotics from the pharmacists’ perspectives in Saudi Arabia	Semi-structured interviews; 20 pharmacists; Community pharmacies in Eastern Province of Saudi Arabia	January-May 2017
Alkadhimi et al., 2020 (Iraq)	Pharmacy Practice (2023: 2,4)	To explore the dispensing practice of antibiotics in community pharmacies in addition to understanding the community pharmacists’ perceptions about dispensing antibiotics without prescription	Semi-structured interviews; 20 pharmacists; Community pharmacies in Baghdad	February 2020
Alkirawan et al., 2022 (The Netherlands)	International Journal of Migration, Health & Social Care (2023: 0,7)	To explore the perspectives and expectations of Syrian refugees in The Netherlands about antibiotic use and prescribing in Dutch primary care	Semi-structured interviews; 12 Syrian refugees; primary care	N/A
Alzard et al., 2024 (Australia)	Journal of the Pediatric Infectious Diseases Society (2023: 2,5)	To explore community pharmacists’ and parents’ experiences, opinions, and knowledge regarding antibiotic use in children and the role of CPs in advocating for antimicrobial stewardship	Semi-structured interviews; 47 pharmacists and 46 parents/caregivers of young patients; Community pharmacies in Melbourne	N/A
Amin et al., 2017 (Egypt)	International Journal of Clinical Pharmacy (2023: 2,6)	To examine factors associated with the unwarranted dispensing of subtherapeutic doses of antibiotics in community pharmacies as part of a cold group or upon direct request from patients among community pharmacy staff	Semi-structured interviews; 9 pharmacists and 6 pharmacy assistants; Community pharmacies in Alexandria	April- December 2016
Anderson et al., 2019 (United Kingdom)	BMJ Paediatrics Open (2023: 2,0)	To assess clinicians’ perspectives on the EEPRIS surveillance information intervention, in order to inform its design (content and delivery)	Semi-structured interviews; 18 GPs and 3 nurse practitioners; urban general practitioner surgeries in a South-West of England city	February-July 2016
Aponte-González et al., 2019 (Colombia)	Pharmacy Practice (2023: 2,4)	To explore the attitudes and motivations associated with the use of antibiotics without prescription	Four focus group interviews; 21 adult people; Bogota	June - July 2016
Arnau-Sánchez et al., 2023 (Spain)	Antibiotics (2023: 4,3)	To explore factors influencing inappropriate use of antibiotics in early infancy from the perspective of the primary care paediatrician	Focus group discussions (FGDs); 25 paediatricians; nine health care areas of the public health care system of the Region of Murcia, Spain	November 2021
Ashdown et al., 2021 (United Kingdom)	BMJ Open (2023: 2,4)	To investigate what factors influence GPs’ decisions in the management of at-risk children with influenza-like illness, particularly in relation to antibiotic prescribing decisions	Semi-structured telephone interviews; 41 GPs; General practices in England	March 2013 - March 2014
Atif et al., 2020 (Pakistan)	Journal of Infection and Public Health (2023: 4,7)	To assess the community pharmacists’ knowledge, perceptions and current practices regarding Antibiotic Stewardship Program	Semi-structured interviews; 15 pharmacists; Community pharmacies in the Bahawalpur district of the Punjab province	October - November 2018
Bergsholm et al., 2023a (Norway)	Exploratory Research in Clinical and Social Pharmacy (2023: 1,8)	To explore how knowledge of antibiotic use is collected and communicated between patients, GPs, and pharmacists, and how patients seek, understand and use available information on antibiotics in adherence to prescribed treatment	Seven focus group interviews; 11 pharmacists (three groups), 13 unspecified physicians and GPs (two groups), and 8 patients (two groups); Norway	October 2020 - January 2021
Bergsholm et al., 2023b (Norway)	Journal of Interprofessional Care (2022: 2,7)	To investigate how pharmacists, GPs and patients position pharmacists in their interactions with patients on antibiotic-related matters in Norwegian pharmacies	Seven focus group interviews; 11 pharmacists (three groups), 13 unspecified physicians and GPs (two groups), and 8 patients (two groups); Norway	October 2020 - January 2021
Biezen et al., 2017 (Australia)	NPJ Primary Care Respiratory Medicine (2023: 3,1)	To explore the views, attitude and practices of primary care providers in the management of RTIs in young children	Semi-structured interviews; 20 GPs, 2 practice nurses, 3 maternal child health nurses and 5 pharmacists; Primary care setting	June 2014 - January 2015
Biezen et al., 2019 (Australia)	BMC Family Practice (2023: 3,2)	To compare GPs and parents’ views on antibiotics for RTIs in young children, exploring barriers and contrasting views	Mix methods - Survey, semi-structured interviews and five focus group interviews; 20 GPs (interviews), and 50 parents and carers of children under the age of five (focus groups); Melbourne	June 2014 -July 2015
Bisgaard et al., 2021 (Denmark)	Antibiotics (Basel) (2023: 4,3)	To explore GPs’ considerations and experiences when managing patients with symptoms of acute lower respiratory tract infections	Semi-structured interviews; 7 GPs; urban and rural locations in the North Denmark Region	January - March 2020
Black et al., 2014 (Qatar)	International Journal of Clinical Pharmacy (2023: 2,6)	To assess pharmacists’ opinions relating to antibiotic utilisation in the community setting	3 focus groups and 2 small group interviews; 22 pharmacists; Community pharmacies and primary care	N/A
Bless et al., 2016 (Switzerland)	PLoS One (2023: 2,9)	To investigate how acute gastroenteritis (AG) and campylobacteriosis are managed, to evaluate how patient's health-seeking behaviour and GPs’ clinical decision-making impact surveillance data and to gather data on the incidence of AG and campylobacteriosis in primary care settings	Semi-structured interviews; 69 GPs; GP practices	May-August 2013
Boaitey et al., 2023 (Australia)	Australian Journal of Primary Health (2023: 1,2)	To explore GPs’ awareness and views about using natural history information when consulting about self-limiting infections, and GPs perceptions and use of the antibiotic chapter resources	Semi-structured interviews; 21 GPs; GP practices on the Gold Coast and Brisbane	September 2021 - April 2022
Boiko et al., 2020 (United Kingdom)	BMJ Open (2023: 2,4)	To investigate how primary care prescribers perceive risk and safety concerns associated with reduced antibiotic prescribing	Semi-structured interviews; 23 GPs, 5 nurse (with prescription certificate), 2 pharmacists; 10 general practices in an urban area and a shire town	January - July 2019
Bordado Sköld et al, 2017 (Denmark)	European Journal of General Practice (2022: 3,4)	To explore (i) GPs’ views of antibiotic treatment failure (ATF) in primary care; (ii) how ATF influences the doctor-patient relationship; and (iii) GPs’ understanding of patients’ views of ATF	Semi-structured interviews; 18 GPs; GP practices in Copenhagen area and Zealand region	August - October 2012
Bosley et al., 2021 (United Kingdom)	Contemporary Nurse (2022: 1,6)	To explore antibiotic prescribing and factors which may influence maternal decision making to seek antibiotics for their young children	Mixed-methods case study consisting of quantitative descriptions of antibiotic prescribing data from general practices and six focus groups; 19 mothers of children under five (focus group interviews); a large UK city in Southern England	Quantitative study: July 2016– 2017 Focus group interviews: N/A
Brisley et al., 2023 (Spain)	Medical Anthropology (2022: 2,3)	To explore the prescription and use of antibiotics in Catalonia from the perspective of GPs, residents of Barcelona, and professionals working on antibiotic stewardship	Ethnographic fieldwork and semi-structured interviews; political documents, 4 GPs, 1 clinical researcher, 1, director of pharmacy, 3 residents of Barcelona (interviews); Barcelona	2018 - 2019
Brookes-Howell et al., 2014 (Poland, United Kingdom, Norway, Spain)	Family Practice (2022: 2,4)	To achieve a deeper understanding of parents’ acceptance, or otherwise, of clinicians’ antibiotic prescribing decisions for children with respiratory tract infections	Semi-structured interviews; 63 parents; primary care settings in the cities of Łódź (n = 16), Cardiff (n = 15), Tromsø (n = 12) and Barcelona (n = 20)	June 2008 - April 2009
Cabral, C., Ingram, J., Hay, A.D. & Horwood, J., 2014 (United Kingdom)	Patient Education and Counseling (2023: 2.9)	To investigate parents' experiences and views of clinician communication during primary care consultations for respiratory tract infections in children under 12	Semi-structured interviews; 30 parents of children who had recently consulted for respiratory tract infections; participants' homes across the UK	February-August 2011
Cabral et al., 2016 (United Kingdom)	Annals of Family Medicine (2022: 5.7)	To understand clinicians’ and parents’ perceptions of communication within consultations for respiratory tract infections in children and what influence clinician communication had on parents’ understanding of antibiotic treatment	60 video recorded consultations and video-elicitations semi-structured interviews; 27 parents and 13 clinicians (9 general practitioners; 3 nurse prescribers; 1 physician assistant) of children aged 3 months to 12 years who presented with RTI; primary care in southwest England	May-December 2013
Colliers et al., 2018 (Belgium)	BMJ Open (2023: 2,4)	To assess antibiotic prescribing and dispensing challenges for GPs and pharmacists in out-of-hours primary care, and to identify context-specific elements that can improve AB prescribing in this setting	Semi-structured interviews; 17 GPs, 1 manager, and 5 pharmacists; a Belgian out-of-hours general practitioners cooperative (GPC) and the pharmacist area covered by the GPC (Antwerp city)	N/A
Colliers et al., 2020 (Belgium)	Antibiotics (Basel) (2023: 4,3)	To explore why and how GPs make antibiotic prescribing decisions	Video-recordings of 160 antibiotic prescribing decision consultations and semi-structured interviews; 21 GPs; General practices at out-of-hours primary care	August - November 2018
Courtenay et al., 2017 (United Kingdom)	BMJ Open (2023: 2,4)	To explore patients’ expectations and experiences of non-medical prescriber-led management of respiratory tract infections (RTIs), to examine whether patient expectations for antibiotics affect the likelihood of receiving them and to understand factors influencing patient satisfaction with RTI consultations	Mixed methods - Questionnaires (120 patients) and follow-up interviews; 22 patients, 16 nurses and 1 pharmacist (referred to as non-medical prescribers); general practices and related communities	August 2014 - November 2015
Courtenay et al., 2019 (United Kingdom)	BMJ Open (2023: 2,4)	To identify the factors that influence nurse and pharmacist prescriber management of respiratory tract infections and to identify the behaviour change techniques to support appropriate prescribing behaviour	Semi-structured interviews; 4 pharmacists and 17 nurses; primary care settings	June - July 2017
Cox et al., 2023 (The Netherlands)	Antibiotics (2023: 4.3)	To investigate experiences, expectations, motivations, and perspectives of patients with UTIs in general practice	Semi-structured online interviews; 14 female UTI patients in general practice; primary care, Netherlands	N/A
Dallas et al., 2020 (Australia)	Family Practice (2022: 2,4)	To explore experiences, perceptions and attitudes of GP vocational trainees and supervisors to delayed antibiotic prescribing for acute self-limiting respiratory tract infections (ARTIs).	Semi-structured telephone interviews; 12 GP trainees and 10 supervisors; General practices (the states of New South Wales, Tasmania and the Australian Capital Territory).	April– September 2018.
Darj et al., 2019 (Bangladesh)	Global Health Action (2023: 2.2)	To explore retail pharmacists’ perceptions of AMR and to encourage them to explain their knowledge and role in the AMR situation in Bangladesh	In-depth, semi-structured interviews; 24 pharmacists; retail pharmacies - Dhaka, Bangladesh.	The course of two months in 2018.
Dempsey et al., 2014 (United States of America)	BMC Family Practice (2023: 3,2)	To identify and understand primary care clinician perceptions about antibiotic prescribing for acute bronchitis	Semi-structured interviews; 12 primary care physicians (speciality unknown) and 1 nurse practitioner; primary care; Boston	N/A
Duane et al., 2016 (Ireland)	BMJ Open (2023: 2,4)	To explore the culture of antibiotic prescribing and consumption in the community for urinary tract infections (UTI) from the perspective of the general practitioners (GPs) and community member	In-depth interviews, and 6 focus groups interviews; 15 GPs (in-depth interviews) and 42 focus group participants/patients; rural and urban locations	2013
Durand et al., 2022 (France)	Journal of the American Pharmacists Association (2023: 2,3)	To explore the perceptions, current practices and interventions of community pharmacists regarding antimicrobial stewardship	Semi-structured interviews; 16 pharmacists; community-based pharmacies in rural areas and cities	February -May 2021
Essilini et al., 2021 (France)	JAC Antimicrobial Resistance (2023: 3,7)	To explore French community pharmacists’ views on antibiotic stewardship (ABS) and antibiotic resistance, their role and current practices, and future opportunities for ABS	Semi-structured interviews; 27 pharmacists; community pharmacies in north-eastern France	May - October 2019
Fletcher-Lartey et al., 2016 (Australia)	BMJ Open (2023: 2,4)	To describe what role patients expectation play in GPs antibiotic prescribing for upper respiratory tract infections	Mixed methods approach using a cross-sectional survey and semi-structured interviews; 32 GPs (interview); Primary care GPs	May - August 2014
Gaarslev et al., 2016 (Australia)	Antimicrobial Resistance and Infection Control (2023: 4.8)	To explore the social and cultural norms surrounding expectations for antibiotics and understand possible communication strategies to decrease patient demand	Mixed methods: cross sectional survey and 4(5) focus group interviews; 21(+) adult citizens; Sydney CBD and Western Sydney	August 2014
Gautham et al., 2024 (India)	BMJ Open (2023: 2,4)	To explore the individual, community and health system-level factors influencing community antibiotic practices in rural West Bengal in India	8 Focus group interviews and in-depth interviews; 98 adult community members (focus groups), 7 teachers, 4 elected village representatives, 2 doctors, 3 social workers and 14 community health workers (interviews); South 24 Parganas district, West Bengal	November 2019 - January 2020
Ghiga and Stålsby Lundborg, 2016 (Romania)	Journal of Pharmaceutical Policy and Practice (2023: 4,2)	To explore the perceptions of Romanian pharmacists, when it comes to the role they play in antibiotic consumption and antibiotic resistance	Semi-structured interviews; 18 pharmacists; 16 different districts and Bucharest	February - March 2015
Ghiga et al., 2023 (Romania)	BMC Primary Care (2023: 3,2)	To increase the understanding of howGPs perceive the phenomenon of antibiotic consumption and antibiotic resistance in Romania, including how they see their roles in this respect	Semi-structured interviews; 12 GPs; rural and urban areas; Romania	September - October 2021
Grigoryan et al., 2022 (United States and Germany)	BMC Womens Health (2023: 4,2)	To understand the emotional experience of women with uncomplicated urinary tract infection	Semi-structured interviews; 65 women (40 from US, 25 from Germany); across the two countries	November - December, 2019
Guo et al., 2021 (Singapore)	BMC Family Practice (2023: 3,2)	To explore processes underpinning decision-making for antibiotic prescribing, by considering doctors’ experiences	Semi-structured interviews; 30 primary care physicians (17 with unknown specialisation and 13 GPs); public and private primary care settings	June 2018 - January 2020
Halls et al., 2017 (United Kingdom)	BMJ Open (2023: 2,4)	To explore parents’ perspectives, concerns and experiences of the management of lower respiratory tract infections in children in primary care	Semi-structured interviews; 23 parents; primary care	January 2013 - March 2015
Hika et al., 2022 (New Zealand)	Antibiotics (Basel) (2023: 4,3)	To explore experiences, perceptions and beliefs that Maori have about antibiotics and the use of antibiotics in regard to acute upper respiratory tract symptoms, and of antimicrobial resistance	Semi-structured interviews; 30 Maori; primary care	N/A
Hoang et al., 2024 (Vietnam)	Critical Public Health (2022: 2,8)	To explore how community pharmacists’ everyday practices are entangled with consumers access to primary healthcare system	Semi-structured interviews; 24 pharmacists; Community pharmacies	April- September 2019
Horwood et al., 2016 (United Kingdom)	British Journal of General Practice (2023: 5,3)	To investigate healthcare professional diagnostic and antibiotic prescribing decisions for children with respiratory tract infections	Semi-structured interviews; 22 GPs and 6 nurses; General practices	N/A
Hu et al., 2024 (United Kingdom)	BJGP Open (2022: 2,8)	To explore community pharmacists’ perceptions and experiences of advising patients on management of acute respiratory tract infections and urinary tract infections, and to explore issues regarding use of over-the-counter medicines	Semi-structured interview; 18 pharmacists; Community pharmacies in London and Oxford	November 2019 - March 2020
Jakupi et al., 2019 (Kosovo)	Pharmacy Practice (2023: 2,4)	To explore to explore the attitudes, experiences and knowledge of users, pharmacists and prescribers towards antibiotics in Kosovo	Semi-structured interviews; 8 patients, 4 pharmacists, 4 primary care physicians (3 GPs and 1 with unknown specialisation); primary care	2015-2016
Jones et al., 2018 (United Kingdom)	BMJ Open (2023: 2,4)	To explore pharmacists’ and pharmacy staff attitudes and experiences around selfcare advice for common infections, antibiotic compliance advice, AMS activities and AMR.	Semi-structured interviews and focus group interviews; 8 GPs, 28 pharmacists, 13 pharmacy staff, 6 representatives from pharmacy organisations in England and Wales, and 2 local stakeholders; Pharmacies, general practices and national organisations	N/A
Kaminsky et al., 2020 (Sweden)	BMC Nursing (2023: 3,1)	To describe nurses’ views of telephone nursing work with callers contacting primary healthcare centres regarding respiratory tract infections	Semi-structured interviews; 12 nurses; primary healthcare	January - February 2014
Khan et al., 2021 (Pakistan)	International Journal of Environmental Research and Public Health (2022: 4,6)	To evaluate the knowledge, attitude, and perception of community pharmacists in Pakistan regarding the nonprescription dispensing of antibiotics and how to improve the rational use of antibiotics	A two-phase mixed-methods study 1. Online questionnaire, a cross-sectional study; 180 community pharmacists; community pharmacy services2.Telephone/online semi-structured interviews; 21 pharmacists; community pharmacy services	August 2019 - March 2020
Khan et al., 2022 (Pakistan)	Frontiers in Pharmacology (2024: 4,4)	To investigate the knowledge, attitude, and practices on antibiotic consumption, antibiotic resistance, and related suggestions among residents of conflicted zones in Pakistan	Semi-structured interviews; 20 patients ; community pharmacies in Pashto	September 2019-January 2020
Knobloch et al., 2021 (United States of America)	American Journal of Infection Control (2023: 3,8)	To identify barriers and facilitators to guideline-concordant prescribing among nurse practitioners prescribing to veterans in an outpatient setting, and to explore perspectives about perceived roles in antibiotic stewardship efforts	Mixed methods design, consisting of quantitative data on nurse practitioners prescribing for diagnosis in which antibiotics are not indicated and individual qualitative semi-structured, interviews and focus group interviews; 14 nurse practitioners, 15 veterans (3 focus groups interviews); outpatient veterans care	November 2019 - January 2020
Kurotschka et al., 2024 (Germany)	BJGP OPEN (2023: 5,3)	To explore the decision making of GPs when managing uncomplicated urinary tract infections in women	Semi-structured, face-to-face interviews; 22 GPs in Bavaria and Baden-Württemberg (southern Germany)	September - December 2019
Laytner et al., 2023 (United States of America)	The Journal of the American Board of Family Medicine (2022: 2,0)	To identify the situations, reasons, and motivations influencing Hispanic patients’ nonprescription use	Semi-structured digitally interviews (second phase of a mixed method study); 35 Hispanic patients; Public clinics in Houston and one private emergency department	May 2020 - October 2021
Lescure et al., 2022 (The Netherlands)	BMC Primary Care (2023: 3,2)	To establish GPs’ and pharmacists’ perceptions, attitudes and experiences regarding the provision of antibiotics to immigrant patients	In-depth, semi-structured interviews; 10 GPs and 5 pharmacists in Rotterdam	November 2018 - August 2020
Lipstein et al., 2019 (United States of America)	Clinical Pediatrics (2023: 1,0)	To understand how parents and physicians make decisions regarding antibiotics and whether a potential associated risk of obesity would alter decisions	8 focus group interviews and individual, semi-structured interviews; 59 parents/ caregivers of children under age 7 years (focus group) and 22 physicians (19 paediatricians; 2 family physicians, 1 medicine-paediatrician (individual interviews)	N/A
Lum et al., 2017 (Australia)	BMC Public Health (2023: 3,5)	To investigate the perspectives, attitudes and behaviours of Australian patients on antibiotic use and antibiotic resistance, to inform national programs for reducing inappropriate antibiotic consumption	Semi-structured interviews; 32 patients; South East Queensland	May - June 2015
Lum et al., 2018 (Australia)	Infection, Disease & Health (2023: 2,7)	To establish the dominant factors influencing GPs decision-making in antibiotic prescribing in the Australian primary healthcare sector	Mixed method design consisting of semi-structured interviews and a Discrete Choice Experiment; 33 GPs; Brisbane and Greater Brisbane, Queensland	September 2015 - October 2016
Mahmoud et al., 2018 (Saudi Arabia)	Biomedical Research (2022: 3,07)	To explore community pharmacists’ views, experiences, and perceptions about antibiotics dispensing without prescription	Semi-structured interviews; 16 pharmacists; community pharmacies in Riyadh, Saudi Arabia	N/A
Manderson, L., 2020 (South Africa)	Humanities & Social Sciences Communications (2023: 3,7)	To explore factors in the provision of health care, health systems and structural factors, and communication between providers and patients that influence the use of antibiotics	Observation study, semi-structured interviews in GP practice and open-ended interviews with stakeholders; 65 patients/parents/guardians, 8 primary care physicians (speciality unknown) , 15 nurse practitioners/senior community nurses, 2 pharmacists; 12 stakeholders; private physicians’ surgeries and community health centres	June - September 2017
Mas-Dalmau et al., 2023 (Spain)	BMC Primary Care (2023: 3,2)	To explore perceptions and attitudes in primary care providers, regarding antibiotic use and different strategies for uncomplicated respiratory tract infections	4 focus group discussions and individual semi-structured interviews; 25 GPs, 1 nurse; primary care centres in Barcelona metropolitan area, Spain	September 2013 - December 2018
McDermott, L., Leydon, G. M., Halls, A., Kelly, J., Nagle, A., White, J. & Little, P., 2017 (United Kingdom)	BMJ Open (2022: 2,9)	To explore perceptions of illness, the decisions to consult and the acceptability of delayed antibiotic prescriptions and self-help treatments for respiratory tract infections	Semi-structured interviews (face-to-face and telephone); 20 patients; Primary care in South of England	N/A
Medina-Perucha et al., 2020 (Spain)	PLoS ONE (2021: 3,7)	To explore the experiences and concerns of service users with acute lower respiratory tract infections, in relation to access to healthcare, antibiotic use and health education in Catalonia	Semi-structured interviews; 29 patients; three primary healthcare centres in Barcelona and one in Tarragona, Catalonia, Spain	April-June 2019
Mortazhejri et al., 2020 (Canada)	BMC Family Practice (2022: 2,9)	To explore how individuals perceive upper respiratory tract infections and how their perceptions influence their self-management, primary care consultation and antibiotic use	Semi-structured telephone interviews; 15 patients; urban/rural family practice in Eastern Ontario, Canada	N/A
Musoke et al., 2023 (Uganda)	Journal of Pharmaceutical Policy and Practice (2023: 4,2)	To explore knowledge and practices related to AMS in private pharmacies in Wakiso district, central Uganda	31 in-depth interviews; 3 clinical officers, 3 midwives, 6 nurses, 5 nursing assistants, 1 pharmacist, 2 enrolled nurses, 9 pharmacy technicians, 1 psychiatric nurse, 1 social scientist; Private pharmacies in Wakiso district, central Uganda	2022
Mustafa et al., 2014 (United Kingdoms)	Annals of Family Medicine (2021: 5,7)	To explore how and why GPs (family physicians) elicit and address patients’ or parents’ expectations for antibiotics	In-depth, face-to-face semi-structured interviews; 20 GPs; South Wales, United Kingdom	October 2010 - April 2011
O'Doherty et al., 2019 (Ireland)	BMC Family Practice (2022: 2,9)	To investigate why GPs continue to prescribe antibiotics for ARTIs despite increasing knowledge of their poor efficacy and worsening antimicrobial resistance	Semi-structured interviews; 13 GPs; General practices (urban and rural settings), Mid-West of Ireland	June - August 2017
Om et al., 2017 (Cambodia)	Antimicrobial Resistance & Infection Control (2023: 4,8)	To explore healthcare seeking behaviour related to obtaining antibiotics and drivers of antibiotic misuse in the Cambodian community	6 focus group discussions and in-depth, individual interviews; 30 nurses (FGD), 35 family members of hospitalised patients, 10 pharmacists (individual interviews); Public hospitals, private pharmacies and community primary healthcare centres in Cambodia	September 2013 - February 2014
Özcebe et al., 2022 (Turkey, Germany, Sweden and the Netherlands)	BMC Primary Care (2023: 3,2)	To explore GPs and pharmacists’ experiences and perspectives on rational antibiotic use among Turkish adults in Turkey and among Turkish migrants in Germany, Sweden, and the Netherlands	In-depth, semi-structured face-to-face/telephone interviews; 21 GPs (family physicians) and 24 pharmacists; community care	2016 - 2017
Peiffer-Smadja et al., 2020 (United Kingdom)	Antibiotics (2023: 4,3)	To explore the views of pharmacy staff and patients on providing or receiving advice for suspected or confirmed urinary tract infections in the community pharmacy setting, and to identify opportunities to enhance the role of community pharmacists in the management of patients	A mixed method study; two surveys and semi-structured interviews; 22 pharmacists; Community pharmacy settings in Newham, London	April 2019-?
Poss-Doering et al., 2020 (Germany)	Antimicrobial Resistance and Infection Control (2023: 4,8)	To foster awareness and understanding of the growing challenge and promote rational antibiotics use for acute, non-complicated and self-limiting infections	Mixed method: A one-time socio- demographic survey and semi-structured telephone interviews; 27 physicians (speciality unknown); Primary care networks in Bavaria and North-Rhine Westphalia	March - June 2018
Raspopovic et al., 2016 (Montenegro)	Medicinski Casopis (Impact Factor unknown)	To reveal factors that influence unduly prescribing antibiotics and the emergence of resistance to antibiotics in primary health care	One focus group interviews; 6 primary care physicians (speciality unknown), 1 paediatrician, 1 pharmacists; Health centre in Danilovgrad, Montenegro	November 2015 - June 2016
Res et al., 2017 (Australia)	Journal of Pharmacy Practice (2023: 2,4)	To explore the role of community pharmacists in the optimisation of antibiotic prescribing and utilisation.	Four focus group interviews; 24 pharmacists; Community pharmacy in the Perth metropolitan area	March - April 2013
Ryves et al., 2016 (United Kingdom)	BMJ Open (2022: 2,9)	To identify GPs views on the use of delayed prescribing, their use of the technique and factors that can enhance or inhibit its use in routine general practice, and to elicit GPs’ views on current prescribing guidelines, and what information would be beneficial if training were to be provided	Semi-structured telephone interviews; 32 GPs; GP practices in England	November 2013
Saleem et al., 2019 (Pakistan)	Family medicine and Community Health (2022: 6,1)	To explore the determinants of AMR and the pattern of antimicrobial dispensing among community pharmacists	In-depth, face-to-face, semi-structured interviews; 12 pharmacists; Community pharmacists in Lahore, Pakistan	October 2017 - January 2018
Saliba-Gustafsson et al., 2019 (Malta)	PLoS ONE (2021: 3,7)	To explore GPs’ understanding of antibiotic use and resistance, and describe their perceived barriers and facilitators to prudent antibiotic prescribing for acute respiratory tract infections in Malta	Face-to-face individual semi-structured interviews; 20 GPs; Public and/or private GP practices in Malta	August - September 2014
Saliba-Gustafsson et al., 2021 (Malta)	PLoS ONE (2021: 3,7)	To explore and describe the perceptions of delayed antibiotic prescription for respiratory tract infections among GPs in Malta	Individual, semi-structured face-to-face interviews; 20 GPs; Public and/or private GP practices in Malta	August - September 2014
Salim and Elgizoli, 2017 (Sudan)	International Journal of Pharmacy Practice (2023: 1,5)	To explore the perspectives of community pharmacists about why they dispense antibiotics without prescription, and to understand their opinions about why they think patients self-medicate	Individual, in-depth face-to-face interviews; 30 pharmacists; Community pharmacies in Khartoum States, Sudan	May - June 2015
Sargent et al., 2017 (Australia)	BMC Family Practice (2022: 2,9)	To identify facilitators and barriers to GPs’ use of delayed prescribing and to gain pharmacists’ and the public’s views about delayed prescribing in Australia	Semi-structured, face-to-face interviews; 18 GPs, 9 pharmacists, 3 pharmacy assistants and 14 patients; the Gold Coast and the Sunshine Coast, Queensland, Australia	February 2014 - July 2015
Sayood et al., 2021 (United States of America)	Journal of the American Pharmacists Association (2023: 2,5)	To determine community pharmacist attitudes towards using a computerised CDS tool to evaluate and manage common complaints to then promote appropriate antibiotic prescribing	In-depth semi-structured telephone interviews; 21 pharmacists; Community pharmacies in Missouri, Illinois, California, Arizona, Utah, Tennessee, and Texas	October 2019 - May 2020
Sharaf et al., 2021 (Qatar)	Antibiotics (2023: 4,3)	To explore barriers of appropriate antibiotic prescription from the physicians’ and pharmacists’ perspectives at primary healthcare centres in Qatar	Five focus groups; 30 primary care physicians (family medicine physicians, GPs, specialists or consultants), 20 pharmacists; Two primary health care centres in Qatar	N/A
Simeoni et al., 2022 (Canada)	BMC Primary Care (2023: 3,2)	To identify potentially modifiable determinants of antibiotic prescribing for patients presenting to primary care with upper respiratory tract infection symptoms	Semi-structured telephone interviews; 20 GPs (family) physicians); Primary care and walk-in clinics in Ontario	March - December 2019
Souto-López et al., 2020 (Spain)	Acta Paediatrica (2023: 2,4)	To explore the parent-related factors underlying antibiotic misuse/overuse and their implication in the development of resistance in the paediatric population	Five focus group interviews; 30 parents of children under 12 years of age; Galicia, north-west Spain	2017
Spicer et al., 2020, (United States of America)	Open Forum Infectious Diseases (2021: 4,42)	To understand whether adult patients viewed antibiotic risk differently and determine whether other antibiotic risks, such as adverse drug events, would be more effective for public health messaging	12 focus groups via telephone; 31 participants (15 parents and 16 adult patients) New York, Rhode Island, Kentucky, Louisiana, Mississippi, Tennessee, West Virginia, Iowa, Nebraska, Utah	March 2017
Stivers, T. & Timmermans, S., 2021 (United States of America)	Social Science & Medicine (2023: 4,9)	To advance the understanding of physician-patient negotiation in the context of acute respiratory infections	Observation study: Two corpora of 68 video recordings of primary care consultations; 30 primary care physicians (speciality unknown); community-based clinics in Southern California	Data collection 1: 2003 - 2004 Data collection 2: 2015 - 2016
Sundvall et al., 2020 (Sweden)	BJGP Open (2023: 5,3)	To explore how opportunities and obstacles for rational antibiotic prescribing were perceived by primary health care centres	Document analysis including 50 reports from primary health care centres in Region Västra Götaland, Sweden	2013-2016
Suy et al., 2019 (Cambodia)	BMJ Global Health (2024: 7,1)	To investigate factors influencing community decisions on purchasing medicines from primary care providers and reasons for using invisible medicine sellers and compare different primary care providers' knowledge of antibiotic use	Seven focus group discussions and individual, semi-structured interviews; 60 community members (FGD), 5 pharmacists, 3 primary care physicians (speciality unknown), 5 medicine sellers, 1 midwife, 1 nurse, 1 unqualified seller, 5 invisible medicine sellers, 4 government health centres staff, 4 community health workers, 6 village leaders (individual interviews); peri-urban districts in Phnom Penh, Cambodia	N/A
Sychareun et al., 2022 (Lao PDR)	BMCPregnancy and Childbirth (2023: 2.8)	To explore perceptions and reported practices of pregnant women and mothers of children under two regarding antibiotic use and resistance	Six focus groups discussions; 55 participants (pregnant women and mothers with children under two years of age) Toulakhom district in Vientiane Province, Lao PDR	September 2019
Thaggard et al., 2023 (New Zealand)	BMC Infectious Diseases (2023: 3,4)	To explore whānau Māori and Pacific people’s knowledge, perceptions, and expectations regarding antibiotic treatment of URTIs with the aim of informing development of educational resources that could build knowledge and skills and reduce the inappropriate prescribing of antibiotics	Six focus group interviews; 47 adult citizens; Auckland (NZ’s largest city) and Taranaki (regional city)	N/A
Tonna et al., 2020 (United Kingdom)	International Journal of Clinical Pharmacy (2023: 2,6)	To explore views and experiences of community pharmacy teams across Scotland on antimicrobial stewardship, activities related to European Antibiotic Awareness Day, and a self-help guide to treating infection	Semi-structured in-depth telephone interviews; 20 pharmacists, 5 pharmacy graduates undertaking a one year internship, 2 pharmacy technicians, 1 medicines counter assistant; Community pharmacies in Scotland	November - December 2016
Torres et al., 2020 (Mozambique)	Pharmacy Practice (2023: 2,4)	To describe the practices and the enablers for non-prescribed antibiotic dispensing in Maputo city, Mozambique	In-depth, face-to-face, semi-structured interviews; 17 pharmacists; Private pharmacies, Maputo city	October 2018 - March 2019
Torres et al., 2023 (Mozambique)	Journal of Public Health: From Theory to Practice (2023: 1,9)	To describe the underlying factors influencing self-medication with antibiotics in Maputo city, Mozambique	Individual, in-depth interviews and two focus group discussions; 32 patients; Pharmacies, Maputo city	October 2018 - March 2019
van der Zande et al., 2019 (United Kingdom)	BMC Family Practice (2022: 2,9)	To understand contextual factors related to GPs’ antibiotic prescribing behaviour in low, high, and around the mean prescribing primary care practices	Semi-structured interviews; 41 GPs; GP practices in a large urban North-West English city	January - June 2018
van Hecke et al., 2019a (United Kingdom)	Journal of Antimicrobial Chemotherapy (2023: 3.9)	To explore parents’ perceptions and understanding of antibiotic use and resistance in the context of their young child with an acute respiratory tract infection and to explore strategies parents would find acceptable to minimise antibiotic resistance for their families	Semi-structured interviews; 23 parents of preschool children; Thames Valley region: Berkshire, Buckinghamshire and Oxfordshire, South-East England	2016–2017
van Hecke et al., 2019b (South Africa)	BMJ Open (2022: 2,9)	To explore the perceptions of primary care providers about prescribing antibiotics for two common infection syndromes, their experiences of existing point-of-care testing, and their perceptions of the barriers and opportunities for introducing new point-of-care testing	Semi-structured interviews; 8 nurses, 4 physicians without specialisation, 11 GPs (family physicians); Publicly funded clinics in the Western Cape Metro district, South Africa	March - April 2018
van Horrik et al., 2024 (The Netherlands)	BJGP Open (2023: 5,3)	To identify barriers and facilitators for applying shared decision making in cystitis management in general practice	Semi-structured interviews; 10 GPs, 7 GP assistants, and 15 patients; general practice	May - October 2022
Williams et al., 2018 (United Kingdom)	Journal of Antimicrobial Chemotherapy (2023: 3,9)	To explore GPs and nurse prescribers’ views on and experiences of prescribing antibiotics for RTIs in primary care out-of-hours services	Semi- structured telephone interviews; 15 GPs and 15 nurses (with prescription certificate); rural and urban primary care out-of-hours services	November 2015 - April 2016
Yates et al., 2018 (United States of America)	BMC Family Practice (2022: 2,9)	To explore elements influencing primary care provider decisions to prescribe antibiotics, identify provider recommendations for interventions to reduce inappropriate antibiotic use, and inform the clinical management of patients in the outpatient environment for infections that do not require antibiotics	Semi-structured interviews; 10 primary care physicians (speciality unknown), 7 advanced care practitioners (nurse practitioners and physicians assistants); primary care	November - December 2016
Zago et al., 2023 (Brazil)	Health Expectations (2023: 3)	To map aspects that shape users' lay knowledge regarding antibiotics use and AMR	Individual in‐depth interviews; 19 adult citizens; Brazil	August - October 2021
Zetts et al., 2020 (United States of America)	BMJ Open (2022: 2,9)	To assess primary care physicians current attitudes towards antibiotic resistance, inappropriate antibiotic prescribing and the feasibility of outpatient stewardship efforts	8 focus group interviews; 26 primary care physicians (family medicine, internal medicine physicians), 26 paediatricians; community care	November - December 2017

The results sections of the publications were coded, and these codes were reorganized to align with the review’s aim (Braun and Clarke, 2006). Initial themes were constructed from the coded material based on similarities and differences. Similar codes were grouped into themes. The themes were reviewed and further developed through a consensual process among the authors, iterating between the constructed themes, the empirical data, and the research question to ensure the themes accurately reflected the empirical material (Braun and Clarke, 2006). Finally, each main theme and sub-theme were defined, refined, named, and reviewed to ensure they were concise and adequately descriptive (Braun and Clarke, 2006). The (sub)themes were narratively described to achieve the study’s aim. For practical reasons, we refer to all non-professional actors as patients. However, we recognize that many participants are not current patients but citizens representing former or potential patients, clients, parents, or others.

## Results

### Characteristics of the studies

In total, 102 publications were included, all published in English (see **Table 4** for details). Of these, 14 publications primarily focused on primary care physicians’, pharmacists’, and nurses’ education of patients about antibiotic use and antimicrobial resistance in primary care settings (Alzard et al., 2024; Atif et al., 2020; Bergsholm et al., 2023a, 2023b; Cabral et al., 2014, 2016; Durand et al., 2022; Essilini et al., 2021; Ghiga and Stålsby Lundborg, 2016; Hu et al., 2024; Knobloch et al., 2021; Manderson, 2020; Musoke et al., 2023; Peiffer-Smadja et al., 2020). The remaining 88 publications addressed primary care physicians’, pharmacists’, and nurses’ education of patients about antibiotic use and antimicrobial resistance in primary care settings as a secondary focus.

The studies were conducted in 38 different countries across all populated continents: the United Kingdom (n=21), Australia (n=11), the USA (n=10), Spain (n=6), the Netherlands (n=5), Pakistan (n=4), Germany (n=4), Sweden (n=3), and Norway (n=3). Additionally, 13 other countries each contributed two publications, and 17 countries were represented by one publication each. Three studies were conducted across multiple countries (Brookes-Howell et al., 2014; Grigoryan et al., 2022; Özcebe et al., 2022).

A majority of the publications (n = 66) used individual semi-structured interviews as a data collection method, conducted either face-to-face or through video/telephone. Eleven publications used focus groups as the only data collection method, one study collected reports/documents as empirical material, and one publication used observations. Fourteen publications combined qualitative methods such as observations, video recordings, documents, and individual and/or focus group interviews. Mixed methods using different quantitative and qualitative data collection methods were used in nine publications.

The studies were all published between 2014 and 2024 and conducted between 2010 and 2022. One study, Stivers and Timmermans (2021) also included a study period from 2003 to 2004. Seventeen of the other publications did not specify their study periods. The publications’ total population included 962 primary care physicians, such as GPs, psychiatrists, surgeons, pediatricians and unspecified physicians, 591 pharmacists, 147 nurses, 1100 (+) patients and 236 others, including stakeholders and other health care professionals.

All selected publications demonstrated appropriate methodological rigor based on the outcomes of the CASP checklist (Critical Appraisal Skills Program, 2018) (**Table 3**).

### Relationships between patients and professionals influenced educational strategies

#### The significance of a robust patient-professional relationship

From the perspectives of primary care physicians, pharmacists, nurses, and patients, a trusting relationship was perceived to create an environment where patients could feel acknowledged and heard (Alzard et al., 2024; Bergsholm et al., 2023a; Brookes-Howell et al., 2014; Dallas et al., 2020; Durand et al., 2022; Ghiga and Stålsby Lundborg, 2016; Hika et al., 2022; Khan et al., 2022; Mustafa et al., 2014; Sargent et al., 2017; Sköld et al., 2017; Souto-López et al., 2020; Spicer et al., 2020; Suy et al., 2019; Thaggard et al., 2023; Zetts et al., 2020). Trust and robustness was crucial when facilitating effective education and guidance regarding antibiotic use, and it reduced patient expectations for antibiotics (Alhomoud et al., 2018; Aponte-González et al., 2019; Bergsholm et al., 2023b; Ghiga and Stålsby Lundborg, 2016; Saleem et al., 2019; Sargent et al., 2017; Simeoni et al., 2022; Zetts et al., 2020). This allowed the professionals to manage patient expectations more effectively, including explaining the reasons for not prescribing antibiotics (Simeoni et al., 2022; Zetts et al., 2020). For instance, both primary care physicians and nurses highlighted the importance of discussing treatment options with the patients’, facilitating a collaborative environment where patients felt their input was valued (Boiko et al., 2020; Guo et al., 2021; Halls et al., 2017). Discussing antibiotic use with patients gave some primary care physicians a sense of control over the situation (Dallas et al., 2020; Guo et al., 2021; Kurotschka et al., 2024). This included discussing different treatments’ potential benefits and AMR risks (Ashdown et al., 2016; Kurotschka et al., 2024). Several primary care physicians perceived these approaches as critical components of the educational role (Ashdown et al., 2016; Guo et al., 2021; Kurotschka et al., 2024).

According to primary care physicians’, pharmacists’ and patients’ view, a solid relationship promoted adherence to prescribed treatments and overall receptiveness to medical advice (Alzard et al., 2024; Guo et al., 2021; Hika et al., 2022; Kurotschka et al., 2024; Lum et al., 2017; Mustafa et al., 2014). Pharmacists also engaged patients in decision-making by asking pertinent questions about physicians’ advice, such as dosing intervals or indications of a specific antibiotic (Bergsholm et al., 2023a). Furthermore, taking into account patients’ previous experiences with medications was reported to foster trust, which emerged as a critical factor in building confidence in prescribing/dispensing decisions (Bergsholm et al., 2023b; Brookes-Howell et al., 2014; Courtenay et al., 2019; Dallas et al., 2020; Guo et al., 2021; Knobloch et al., 2021; van der Zande et al., 2019; Zetts et al., 2020). Some pharmacists highlighted that informal interactions fostered rapport, increased awareness of antibiotic use and resistance (Durand et al., 2022), and enhanced their accessibility and role in community healthcare through valuable information and free counselling during medication dispensing (Alhomoud et al., 2018). Primary care physicians and pharmacists also noted that a solid relationship could help manage patient dissatisfaction, even when treatments did not meet patients’ expectations (Kurotschka et al., 2024; Ghiga et al., 2023; Simeoni et al., 2022). Explaining why antibiotics were not prescribed while acknowledging patients’ experiences helped build stronger relationships.

#### Various strategies for facilitating effective communication

Several primary care physicians and nurses emphasized the significance of practical communication skills in explaining treatment decisions, particularly the decision not to prescribe antibiotics (Bergsholm et al., 2023a; Courtenay et al., 2017; Sköld et al., 2017). Effective communication involved using precise language, providing clear information, and addressing potential misunderstandings, regardless of the healthcare professional’s background (Alkirawan et al., 2022; Bergsholm et al., 2023b; Fletcher-Lartey et al., 2016; Lum et al., 2017). Various strategies were reported, such as creating a more inclusive and supportive environment, bridging gaps in patient understanding, and using informal language (Bergsholm et al., 2023b; Guo et al., 2021). Addressing language barriers and providing both verbal and written information were also perceived as effective strategies for improving communication (Fletcher-Lartey et al., 2016; Laytner et al., 2023).

Primary care physicians frequently used clinical tools, such as C-reactive protein tests, to explain why antibiotics were unnecessary (Lescure et al., 2022; Özcebe et al., 2022). Pharmacists supported reduction of antibiotic misuse by providing clear instructions on dosages, explaining the risks of misuse, and offering non-antibiotic alternatives (Jones et al., 2018; Manderson, 2020; Om et al., 2017; Özcebe et al., 2022; Saleem et al., 2019; Sayood et al., 2021). Some pharmacists also asked follow-up questions to assess symptom severity and to guide or advise patients to see a physician (Mahmoud et al., 2018; Sayood et al., 2021). Some experienced primary care physicians also developed strategies such as ‘preparing the ground’, which involved taking a comprehensive history, conducting thorough examinations, and communicating decisions empathetically (Lum et al., 2018). Medical histories and clinical examinations also played a crucial role in explaining why antibiotics were not prescribed, demonstrating that decisions were made with patients’ best interests in mind while fostering trust and managing expectations (Lum et al., 2018; Manderson, 2020).

Primary care physicians, pharmacists and nurses emphasized the risks associated with overusing antibiotics, storing leftover medications, self-medicating, and stopping treatment prematurely. They also reassured patients about viral illnesses and normalized infections and encouraged self-management to reduce unnecessary antibiotic use (Anderson et al., 2019; Biezen et al., 2017; Boaitey et al., 2023; Boiko et al., 2020; Saliba-Gustafsson et al., 2021; Yates et al., 2018). Many primary care physicians and nurses viewed running commentary and acknowledging patients’ illnesses during consultations as crucial to reduce unnecessary antibiotic use (Mustafa et al., 2014; Williams et al., 2018). This was perceived as an important help to adjust patients’ preconceived notions about the necessity of antibiotics (Mustafa et al., 2014). Both primary care physicians and pharmacists emphasized the absence of bacterial symptoms (Bergsholm et al., 2023b; Colliers et al., 2020; Knobloch et al., 2021; Cabral et al., 2016; Mustafa et al., 2014; Sköld et al., 2017; Özcebe et al., 2022), which several patients recognized as a valuable effort to educate them and reinforce trust in the decision-making process (Brookes-Howell et al., 2014; Cabral et al., 2014).

Some primary care physicians used therapeutic guidelines on antibiotic use. However, more of them had had negative experiences using guidelines during the consultation, fearing that patients might judge them and believe they were unsure how to treat the condition (Boaitey et al., 2023). In contrast, patients reported a lack of sufficient ‘safety-netting advice,’ indicating that healthcare professionals did not provide enough information (Alhomoud et al., 2018; Alkadhimi et al., 2020; Alzard et al., 2024; Boiko et al., 2020; Colliers et al., 2020; Horwood et al., 2016; Kurotschka et al., 2024). Specifically, patients often felt the information was inadequate regarding the details of their infection, the rationale for not prescribing antibiotics, and the manner in which the information was conveyed (Bergsholm et al., 2023b; Cabral et al., 2014; Souto-López et al., 2020).

#### Different priorities and wishes for treatment

From primary care physicians’ perspectives, while empowered patients were more motivated to engage with and follow the information provided at the pharmacy (Alzard et al., 2024; Bergsholm et al., 2023a), some of them perceived that this level of involvement occasionally undermined trust in their professionality (Mustafa et al., 2014). However, in episodic care settings, where primary care physicians often lacked an established rapport with patients, time constraints and limited access to patient history further challenged their ability to confidently avoid unnecessary antibiotic prescriptions (Ryves et al., 2016; Simeoni et al., 2022). The unfamiliarity with the patients hindered effective patient education about the risks of antibiotics, often leading to a greater likelihood of issuing an antibiotic prescription (Colliers et al., 2020; Duane et al., 2016; Ryves et al., 2016; Simeoni et al., 2022). Moreover, the interpretation of patient histories, symptoms, and test results varied among primary care physicians, reflecting individualized strategies. Some primary care physicians also found it difficult to explain their antibiotic treatment choices, as these decisions were often based on instinct (Bisgaard et al., 2021).

Several primary care physicians, pharmacists and nurses reported that patients often expected antibiotics as a quick fix (Biezen et al., 2019; Bisgaard et al., 2021; Fletcher-Lartey et al., 2016; Horwood et al., 2016; Manderson, 2020; Res et al., 2017; Sharaf et al., 2021; van der Zande et al., 2019). Managing these multifaceted pressures required a range of strategies, with both primary care physicians and pharmacists relying on patient education to address misconceptions about antibiotic use. However, several primary care physicians, pharmacists and nurses also yielded to patient demands, especially when faced with persistent pressure or difficult consultations to avoid conflict (Biezen et al., 2017; Black et al., 2014; Courtenay et al., 2019; Fletcher-Lartey et al., 2016; Kaminsky et al., 2020; Kurotschka et al., 2024; Lum et al., 2018; Manderson, 2020; Mahmoud et al., 2018; Musoke et al., 2023; Res et al., 2017; Ryves et al., 2016; Saliba-Gustafsson et al., 2021). Primary care physicians, pharmacists and nurses often assumed that patients expected antibiotic prescriptions during consultations (Biezen et al., 2017; Boiko et al., 2020; Dallas et al., 2020; Kaminsky et al., 2020; Ryves et al., 2016; Saliba-Gustafsson et al., 2021; Sargent et al., 2017). While some professionals stated to be unaffected by this pressure (Saliba-Gustafsson et al., 2021), primary care physicians and nurses expressed feeling pressured to prescribe antibiotics for infections they did not consider requiring treatment (Arnau-Sánchez et al., 2023; Boaitey et al., 2023; Courtenay et al., 2019; Duane et al., 2016; Hu et al., 2024; Kurotschka et al., 2024; Lum et al., 2018; Mustafa et al., 2014; O'Doherty et al., 2019; van der Zande et al., 2019). Pharmacists reported similar challenges (Mahmoud et al., 2018; Jones et al., 2018), noting that patients frequently pressured pharmacists to dispense antibiotics, sometimes without a prescription. However, as Manderson (2020) highlighted, patients did not always seek antibiotics, indicating that healthcare professionals sometimes misjudged patients’ needs or failed to recognize the importance of patient education.

#### Social position made a difference

Power asymmetries in healthcare interactions were reported as an essential factor that affected both trust and robustness of the patient-professional relationship. Factors such as patient age, cultural background, comorbidities, and symptom severity were stated by professionals to contribute to increased antibiotic prescribing in, for example, episodic care settings (Simeoni et al., 2022). Language and cultural barriers complicated the efforts to educate patients about proper antibiotic use, especially in multicultural settings where communication challenges were common (Colliers et al., 2018; Hu et al., 2024; Lescure et al., 2022; Peiffer-Smadja et al., 2020). Primary care physicians often found it challenging to explain the importance of appropriate antibiotic use, mainly when they encountered patients who had poor language skills, low literacy or cultural differences (Duane et al., 2016; Fletcher-Lartey et al., 2016; Lescure et al., 2022). Primary care physicians, pharmacists and nurses acknowledged that specific training in handling difficult situations and patient conversations could enhance communication and reduce misunderstandings (Ashdown et al., 2016; Biezen et al., 2017; Guo et al., 2021; Lum et al., 2018; Mustafa et al., 2014; Poss-Doering et al., 2020; Raspopovic et al., 2016; Res et al., 2017).

From the patients’ perspectives, a lack of commitment or attentiveness from primary care physicians often undermined trust (Cabral et al., 2014; Grigoryan et al., 2022; Zago et al., 2023). Patients expressed a sense of distrust stemming from the perceived lack of personal commitment from some primary care physicians, who appeared uncaring and inattentive during consultations, marked by minimal eye contact and engagement (Cabral et al., 2014; Zago et al., 2023). Additionally, some pharmacists felt that asking too many diagnostic questions was undesirable, as it could make patients perceive them as uncertain or unqualified (Hoang et al., 2024). In some cases, from patients’ perspective, primary care physicians responded to patients’ questions with dismissive remarks, such as questioning patients’ desire for more information (Gautham et al., 2024). Moreover, an absence of explicit discussion around repeated antibiotic prescriptions by primary care physicians could lead to patients’ distrust (Halls et al., 2017; Hika et al., 2022; Lum et al., 2017). This distrust could sometimes hinder patients’ receptiveness to the information provided (Bergsholm et al., 2023a). Some patients trusted pharmacists more than primary care physicians, viewing pharmacists as impartial, affordable healthcare professionals and the most knowledgeable in medication-related matters (Alhomoud et al., 2018; Mortazhejri et al., 2020). However, other patients perceived pharmacists more as ‘shopkeepers’ than qualified healthcare professionals, which could undermine their role in antibiotic stewardship (Darj et al., 2019).

### Organizational structures challenged professionals in guiding and educating patients

#### Time is money, and vice versa

Primary care physicians, pharmacists, nurses, and patients recognized the urgent need for systemic changes to enhance antibiotic stewardship (Black et al., 2014; Courtenay et al., 2019; Horwood et al., 2016; Manderson, 2020). The practical challenges faced by healthcare professionals in primary care settings significantly affected their ability to effectively educate patients about antibiotic use and AMR (Bosley et al., 2021; Courtenay et al., 2019; Guo et al., 2021; Manderson, 2020; Mas-Dalmau et al., 2023; Peiffer-Smadja et al., 2020; Sharaf et al., 2021; Williams et al., 2018; Yates et al., 2018). These challenges primarily stem from a lack of resource allocation within the healthcare system and time constraints, which hindered the implementation of practices aimed at improving the management of antibiotic prescribing and dispensing (Biezen et al., 2019; Courtenay et al., 2019; Mas-Dalmau et al., 2023; Peiffer-Smadja et al., 2020).

In time-pressured environments, many primary care physicians perceived shared decision-making as too time-consuming, particularly when a more significant number of treatment options needed to be discussed (Alkadhimi et al., 2020; Alzard et al., 2024; Bergsholm et al., 2023a; van Horrik et al., 2024). Time pressures were particularly intense during peak consultation periods or at the end of the week, restricting educational opportunities for physicians and patients (Guo et al., 2021; Kurotschka et al., 2024). Moreover, in some countries such as South Africa, long journeys to clinics and crowded conditions impeded patients’ access to follow-up care and proper education, leading to increased antibiotic use (van Hecke et al., 2019b). Often primary care physicians and nurses managed a high volume of patients each day, which forced them to rely on providing written information to save time during consultations. Nevertheless, some healthcare professionals expressed concern that offering only written information may limit the effectiveness of the educational message reaching patients (Boiko et al., 2020). Furthermore, from nurses’ perspective, time constraints during consultations often led to a focus on advising patients to take prescribed antibiotics without adequately addressing essential topics such as antibiotic resistance or self-care (Manderson, 2020). Patients often perceived rushed consultations as unhelpful, leading them to struggle with understanding essential treatment details such as dosage, duration, and appropriate antibiotic use (Cox et al., 2023; Gautham et al., 2024; Zago et al., 2023).

Less experienced professionals and those working in high-pressure environments were particularly prone to over-prescribing antibiotics (Ryves et al., 2016; Sundvall et al., 2020). Some primary care physicians and nurses opted to prescribe antibiotics as a quicker solution than not doing it, especially when they were running behind schedule or facing a high volume of patients (Biezen et al., 2017, 2019; Brisley et al., 2023; Guo et al., 2021; Kurotschka et al., 2024; Lescure et al., 2022; van der Zande et al., 2019). Discussions about risks and benefits of antibiotics were often limited, and some patients reported that primary care physicians did not always provide essential information regarding the administration of antibiotics or potential adverse reactions (Lum et al., 2017; Manderson, 2020; Mas-Dalmau et al., 2023; Mortazhejri et al., 2020). This lack of education left patients dissatisfied with the consultation and uncertain about when antibiotics were appropriate, contributing to confusion and frustration (Brookes-Howell et al., 2014; Gaarslev et al., 2016; Halls et al., 2017; Laytner et al., 2023; Lipstein et al., 2019; Sychareun et al., 2022; van Hecke et al., 2019a). Pharmacists often faced pressure to provide quick solutions after doctor consultations, leaving little time to assess antibiotic treatments or educate patients on proper use (Alzard et al., 2024). This challenge was especially acute in low-resource settings, where limited healthcare access led patients to rely on pharmacists or informal sources, prioritizing rapid dispensing over patient education (Suy et al., 2019).

The structure of the healthcare system, particularly in private sector settings, introduced another layer of complexity to antibiotic stewardship. A notable conflict existed between financial incentives and the goal of appropriate antibiotic use. Pharmacists often balanced patient numbers and sales targets, which sometimes led to inappropriate dispensing and prescribing of antibiotics, hence prioritizing profitability over patient education (Alkadhimi et al., 2020; Alhomoud et al., 2018; Musoke et al., 2023; Saleem et al., 2019; Salim and Elgizoli, 2017). The commercial pressure to increase sales, therefore, undermined their ability to focus on patient education and AMR prevention (Alkadhimi et al., 2020; Amin et al., 2017; Salim and Elgizoli, 2017; Torres et al., 2020). In some primary care practices with more resources, such as extended consultation times and triage systems, physicians could reduce unnecessary antibiotic prescriptions by educating patients about alternative treatments. However, in some private clinics, the pressure of paid consultations made it challenging for primary care physicians to refuse requests for antibiotics. They felt obligated to provide ‘value’ to private patients who paid a premium, further complicating efforts to educate patients about appropriate antibiotic use (O'Doherty et al., 2019).

#### Technical and educational tools at stake

Several primary care physicians, pharmacists, and nurses believed that handouts and posters could effectively educate patients about unnecessary antibiotics for acute bronchitis. However, some remain skeptical about their effectiveness (Dempsey et al., 2014; Tonna et al., 2020). Pharmacists pointed out that well-intentioned awareness campaigns, such as European Antibiotic Awareness Day, often fail due to information overload, which hinders prescribers and pharmacists from delivering clear and impactful educational messages (Tonna et al., 2020). Patients expressed a desire for more comprehensive information, highlighting that gaps in communication from healthcare professionals lead to changes in how antibiotics are prescribed (BrookesHowell et al., 2014). Additionally, patients wanted primary care physicians to take a more proactive role in antibiotic stewardship (Lum et al., 2017). In contrast, some primary care physicians viewed their involvement in antibiotic stewardship as non-essential, feeling compelled to prescribe (Ghiga et al., 2023) and influenced by the limited use of guidelines (Arnau-Sánchez et al., 2023).

Several primary care physicians refrained from using decision aids to educate patients, citing concerns that these tools might prolong consultations and disrupt their schedules (Boaitey et al., 2023). They also acknowledged the limited education patients receive regarding antibiotics and antimicrobial resistance. To address this gap, they advocated for broader public education efforts, such as school programs, media campaigns, and other community initiatives, to enhance patient awareness on these critical topics (Arnau-Sánchez et al., 2023; O'Doherty et al., 2019; Özcebe et al., 2022). Digital tools were often limited by patients’ lack of digital literacy (Lescure et al., 2022). Existing educational materials were reported not to meet patients’ specific needs. Furthermore, insufficient educational materials tailored to specific patient groups further restricted effective communication and patient education among primary care physicians, pharmacists, and nurses (Fletcher-Lartey et al., 2016; Kaminsky et al., 2020; Manderson, 2020; Sharaf et al., 2021). The absence of adequate IT infrastructure, such as electronic links between physicians and other healthcare professionals, also contributed to defensive prescribing practices (Saliba-Gustafsson et al., 2021). To address this issue, some primary care physicians utilized resources like ‘Choosing Wisely’ pamphlets, which support non-antibiotic alternatives and help reassure patients that their symptoms are being taken seriously (Simeoni et al., 2022). However, some pharmacists pointed out that outdated guidelines complicated their ability to stay informed about antimicrobial treatments, leading them to rely on various online platforms, like Google and Medscape, or to consult drug leaflets (Jakupi et al., 2019; Musoke et al., 2023).

#### Lack of collaboration - a professional hierarchy

Pharmacists emphasized the importance of correct antibiotic use and education in combating antimicrobial resistance (Alhomoud et al., 2018; Alzard et al., 2024; Bergsholm et al., 2023b; Peiffer-Smadja et al., 2020; Res et al., 2017; Sayood et al., 2021). However, pharmacists often encountered patients seeking clarification on issues not fully explained during consultations with primary care physicians. These issues included the reasons for delayed prescriptions and the appropriate dosage and timing of antibiotics (Bergsholm et al., 2023b; Jakupi et al., 2019; Sayood et al., 2021). Consequently, many pharmacists viewed themselves as crucial in educating patients about antibiotic use. They frequently served as the first point of contact for advice on proper usage, potential side effects, and treatment adherence.

Additionally, socioeconomic factors greatly influenced how pharmacists educated patients about antibiotics. Financial constraints often led low-income patients to request fewer antibiotics or seek them without a prescription. Some pharmacists noted that these patients frequently turn to them for advice instead of consulting a physician (Saleem et al., 2019; Salim and Elgizoli, 2017). However, pharmacists’ ability to educate patients was often hindered by limited access to patients’ medical histories, which prevented them from making well-informed decisions about the appropriateness of prescribed antibiotics (Atif et al., 2020; Jones et al., 2018; Khan et al., 2021; Peiffer-Smadja et al., 2020; Sayood et al., 2021). Additionally, many pharmacists encountered communication barriers with primary care physicians, which further limited their ability to support antibiotic stewardship initiatives (Atif et al., 2020; Khan et al., 2021). In contrast, nurses had limited influence on prescribing decisions, as physicians held the final authority in cases such as managing respiratory tract infections. However, nurses frequently encouraged patients to ask their primary care physician about the rationale behind antibiotic prescriptions and offered guidance on managing symptoms without the use of antibiotics (Biezen et al., 2017). From the perspective of primary care physicians, on the other hand, pharmacists’ educational advice regarding antibiotics could conflict with their own guidance, potentially confusing patients (Bergsholm et al., 2023a). Several primary care physicians expressed concerns that pharmacists might exceed their role by providing information beyond what was discussed during consultations. They preferred that pharmacists focus on dispensing medications rather than engaging in clinical discussions with patients (Bergsholm et al., 2023b).

#### Delayed prescription as a tool to balance all demands

When time constraints, follow-up appointments, and collaborative relations were challenging, primary care physicians often felt pressured to prescribe antibiotics as a precaution (Manderson, 2020; Ryves et al., 2016). The practice of issuing delayed prescriptions, in which healthcare professionals such as primary care physicians or nurses provide a prescription for antibiotics but advise patients to wait a specified period before filling it, has become a common yet complex approach (Biezen et al., 2017; Lum et al., 2017, 2018; McDermott et al., 2017; O'Doherty et al., 2019; Poss-Doering et al., 2020; Ryves et al., 2016; Saliba-Gustafsson et al., 2019; Sargent et al., 2017; van der Zande et al., 2019). Both primary care physicians and nurses reported that delayed prescriptions aimed to encourage patients to follow medical advice, thereby granting them greater control over their treatment plans (Boiko et al., 2020; Dallas et al., 2020; Duane et al., 2016; Fletcher-Lartey et al., 2016; Horwood et al., 2016; Lum et al., 2018; Ryves et al., 2016; Saliba-Gustafsson et al., 2019; Sargent et al., 2017).

Variations in prescribing practices among primary care physicians often resulted in patients receiving conflicting advice, making it harder to understand when antibiotics are necessary (Lum et al., 2018; McDermott et al., 2017). Some primary care physicians noted that inconsistent messaging confuses patients and limited opportunities to educate them about respiratory infections (Ryves et al., 2016). Delayed prescriptions or granting patients complete decision-making autonomy were often suggested for specific individuals, based on their ability to understand the strategy (Mas-Dalmau et al., 2023; Ryves et al., 2016; Saliba-Gustafsson et al., 2019). However, many patients expressed discomfort with deciding whether to use antibiotics, preferring to have the physician make that decision instead (Lum et al., 2017), indicating that these strategies were not always effective or productive.

Some primary care physicians acknowledged that they prescribed delayed antibiotics despite weak evidence and concerns regarding misuse or the potential for missing severe infections (Lum et al., 2018; Ryves et al., 2016; Sargent et al., 2017). Additionally, some pharmacists dispense antibiotics without a prescription when patients requested them by name, assuming these patients were knowledgeable about their appropriate use (Mahmoud et al., 2018). Moreover, several primary care physicians pointed out that delayed prescriptions could lead to inappropriate antibiotic use, with patients either storing antibiotics for future use or taking them immediately (Sargent et al., 2017). Nevertheless, diagnostic uncertainty and lack of time led many primary care physicians to prescribe antibiotics ‘just in case’ rather than educating patients on appropriate usage (Biezen et al., 2019; Saliba-Gustafsson et al., 2021; Sayood et al., 2021). Consequently, delayed prescriptions were viewed as a strategy to alleviate professional insecurity and avoid the risk of neglecting to prescribe antibiotics for severe infections (Dallas et al., 2020; Lum et al., 2018; O'Doherty et al., 2019; Ryves et al., 2016; Saliba-Gustafsson et al., 2021; Sargent et al., 2017).

## Discussion

The discussion will focus on three main findings: the importance of relationships between healthcare professionals and patients in facilitating successful patient education about antibiotic use and AMR, the structural challenges that often hindered healthcare professionals from providing detailed education to patients, and the use of delayed prescriptions to balance the improvement of AMR stewardship and met patients’ expectations for antibiotic treatment.

The results showed that strong relationships between healthcare professionals and patients are vital for effective patient education on antibiotic use and AMR. Trust and effective communication were consistently identified as key in ensuring patients feel understood and informed. Primary care physicians, pharmacists, and nurses each play distinct roles in fostering trust, which could encourage patients to follow advice regarding antibiotics. When patients trusted healthcare professionals, they were more likely to accept not receiving unnecessary antibiotics. These findings contribute to the existing literature (Gulliford et al., 2021; Jorgoni et al., 2022) by emphasizing the importance of relationship-building as a central component of antibiotic stewardship rather than focusing solely on medical interventions. This trust-building process is essential in overcoming patient expectations for antibiotics and can significantly influence patient engagement in antibiotic stewardship efforts. Moreover, actively involving patients in conversations about antibiotic use and respecting their preferences can foster a sense of shared decision-making, which is associated with increased patient engagement and better health outcomes (Elwyn et al., 2012; Santana et al., 2018). Such a perception aligns with the idea about person-centered care, which emphasizes the importance of acknowledging patients’ concerns and experiences to build trust (Ekman and Swedberg, 2022; Ridd et al., 2009; Santana et al., 2018). However, the findings also revealed significant challenges in building trust, especially in the context of power dynamics within healthcare settings. The findings indicated that the hierarchical nature of healthcare interactions, where physicians and pharmacists hold positions of authority, could hinder open communication with patients. This power imbalance was found to affect both the trust patients have in healthcare professionals and the effectiveness of educational efforts. In line with Bourdieu’s concept of cultural and symbolic capital (Bourdieu, 1984), the study’s findings suggest that healthcare professionals’ authority can create an asymmetry in patient interactions, reducing opportunities for shared decision-making and undermining the patient’s active role in their treatment. This contrasts with the person-centered approach that the study highlights as being essential for better patient engagement in AMR stewardship. The current study not only confirmed the importance of trust but also revealed how structural and power dynamics within healthcare settings, rooted in the biomedical perspective, may limit the achievement of successful patient education and antibiotic stewardship. These findings resonate with Foucault’s concept of the ‘medical gaze,’ which suggests that the dominance of healthcare professionals’ authority often reframes patient narratives to fit within a biomedical framework, overlooking non-biomedical dimensions of their experiences (Foucault, 2003). Such power dynamics can reduce patients’ agency, thereby affecting the trust required for successful patient education on antibiotics and AMR. In healthcare contexts, patient encounters tend to be more ‘medical/professional-oriented’ than ‘patient/person-oriented’ as they prioritize diagnosing and prescribing over a holistic approach, leading to an asymmetric power structure in primary healthcare (Misselbrook, 2013; Glasdam et al., 2020).

The results showed that pharmacists who adopted informal communication styles were more successful in building relationships with patients, where inconsistent communication between primary care physicians and pharmacists complicated these dynamics. This inconsistency in information exchange regarding antibiotic treatments could undermine patients’ confidence, creating confusion about treatment plans and antibiotic use. Previous literature suggests that this could stem from differing professional positions, roles and responsibilities, and a lack of collaboration between healthcare professionals, highlighting the need for a unified approach to antibiotic stewardship (Balea and Glasdam, 2024; Reeves et al., 2017). Moreover, in line with previous literature, collaboration is embedded in hierarchical structures (Essex et al., 2023). The issue of perceived power asymmetry raises questions about whether healthcare professionals always recognize the impact of their authority on patient interactions. Previous literature shows that failing to address patients’ concerns may diminish trust (Epstein and Street, 2011). This could explain why some patients were less receptive to educational messages about antibiotic use and AMR stewardship, as highlighted in our findings. This study is, however, limited by its lack of focus on patients’ attitudes, knowledge levels, and cultural values. Stewart et al. (2022) argue that patient adherence is influenced by both motivation and ability. Some studies highlight that many young people are inadequately informed about antibiotic treatment and antimicrobial resistance (AMR), often perceiving antibiotics as a universal remedy and demonstrating limited understanding of the differences between viral and bacterial infections (Crago et al., 2022; Hawking et al., 2017). Conversely, other studies suggest that attitudes towards antibiotic treatments and AMR prevention are not necessarily age-dependent (e.g., Zaykova et al., 2022) but are instead shaped by cultural and social factors (Dionisio et al., 2023; Minnssen et al., 2020). This underscores the need for future research to adopt relational perspectives on antibiotic treatment and AMR stewardship, focusing on interactions between healthcare professionals and patients from both perspectives.

The results mainly focused on primary care physicians working as primary care physicians’ and pharmacists’ patient education about antibiotics and AMR. The literature searches did not identify any publications that addressed nurses’ patient education about antibiotic treatment or AMR stewardship in home care and nursing home settings, which are significant areas within primary care. In primary care, including nursing homes, nurses meet patients with infections, treated with antibiotics or not (Alberg et al., 2017; Tark et al., 2020). The current review only found studies about nurses’ patient education concerning prescription. However, follow-up education during treatment and AMR preventive initiatives may also be important tasks for nurses in primary care, which calls for future studies. Nurses, among other healthcare professionals, play a major role when it comes to the spreading of infections and the development/stewardship of AMR (Glasdam et al., 2021; Singh et al., 2022), as they act as carriers and thereby transmit resistant bacteria to patients (Fracarolli et al., 2017; de Oliveira et al., 2012).

Furthermore, the results suggested that structural challenges, such as time pressures, heavy workloads, and the commercial nature of pharmacies and general practices, often hinder healthcare professionals from providing detailed patient education, leading to gaps in understanding of both treatment and prevention of AMR. Time constraints are among the most significant barriers healthcare professionals face today (Kasse et al., 2024; Lansink et al., 2024), directly impacting professionals’ ability to provide comprehensive patient education about antibiotic use (Bosley et al., 2018). As the current results showed, primary care physicians often managed large numbers of patients daily, leaving insufficient time for thorough conversations about whether antibiotics were necessary for a given condition. Consequently, patients may not fully understand why antibiotics are being withheld, leading to frustration or demands for unnecessary prescriptions. The desire to increase patient turnover, in line with commercial goals, was evident in the current results, limiting the duration and quality of consultations. Pharmacists in commercially driven settings faced the dual challenge of high patient demand and the pressure to maintain profitability. While pharmacists were tasked with counselling patients on safe medication use, including antibiotics, their ability to provide detailed guidance was compromised by the need to meet sales targets. The prioritization of profitability over patient care may lead to less time spent educating patients about the risks of antibiotic misuse (Balea and Glasdam, 2024). In such profit-oriented environments, the commercial pressures conflict with the professional responsibility as healthcare professionals may feel driven to maximize income at the expense of providing adequate patient education (Balea and Glasdam, 2024; Saleh et al., 2021). The pursuit of financial success, therefore, further complicates efforts to combat AMR, as pharmacists and primary care physicians may be incentivized to prioritize sales/number of consultations over detailed patient education.

Moreover, the global shortage of primary care physicians exacerbates this problem. In many parts of the world, the demand for healthcare professionals far exceeds supply, resulting in overwhelmed primary care physicians who must see more patients in less time (Shen et al., 2020; Velgan et al., 2023). This shortage amplifies the pressures to balance patient care with business success, particularly in private practices where primary care physicians must manage the financial sustainability of their operations. The drive to earn more money can increase patient throughput, limiting the time available for important discussions about antibiotic use and AMR prevention.

These findings underscore the persistent tension between the demands of efficient healthcare delivery and the ethical responsibility to provide thorough patient care, which can lead to unnecessary prescriptions and contribute to the AMR crisis (Cutrell and Sanders, 2024; Pokharel et al., 2024). The findings highlight a central challenge in modern healthcare: balancing the commercial aspects of healthcare provision with the moral obligation to uphold the principles of the Hippocratic Oath as interpreted in the Geneva Declaration (World Medical Association, 2017) and the medical ethical principle, especially the duty to ‘do no harm’ (Varkey, 2021). This tension is particularly evident in the context of AMR and the roles of primary care physicians and pharmacists in ensuring proper antibiotic stewardship. At the heart of this dilemma is also the desire of healthcare professionals to achieve financial success in their business (Noor et al., 2022). The conflict between profitability and patient care invites reflection on how healthcare systems might address the balance so as not to harm the patient in the first instance, and society at large by contributing to the development of AMR. However, the current findings also revealed that some primary care physicians and pharmacists were able to educate patients under the existing structural framework.

The results revealed the use of delayed prescriptions to balance the improvement of AMR stewardship and meeting patients’ expectations for antibiotic treatment. Traditionally, physicians are the primary decision-makers and prescribers in antibiotic therapy (Carlsson et al., 2023). However, findings revealed a role inversion, shifting the basis of antibiotic stewardship from the expert, alias the physician, to the patient, thereby diminishing the professional autonomy, pointing to a form of de-professionalization of medicine (Engelhardt, 2002). This suggests that, in their efforts to maintain professional authority, physicians often rely on personal judgement, allowing external factors, such as patient preferences and concerns about risks of bacterial infection, to influence their decisions regarding antibiotic stewardship (Kasse et al., 2024; Lansink et al., 2024). This trend reflects broader challenges within healthcare, such as diagnostic uncertainties and time constraints, which can complicate the decision-making processes. While delayed prescriptions aim to reduce unnecessary antibiotic use, they also raise concerns about whether this approach may undermine professional responsibility and the authority of healthcare professionals in ensuring optimal antibiotic stewardship (Mcleod et al., 2024).

Davari et al. (2018) state that, in practice, physicians’ prescribing decisions are influenced by numerous factors, including clinical uncertainties, comorbidities, and patient expectations. When faced with unclear diagnoses or fear of complications, physicians may shift from clinical guidelines to a more individualized approach, as Md Rezal et al. (2015) demonstrate, despite acknowledging guidelines, many physicians deviate due to pressures like patient demands or diagnostic uncertainty. Similarly, McCullough et al. (2017) found a gap between guideline recommendations and actual prescribing rates, particularly for respiratory infections. Not all healthcare settings universally provide or follow detailed guidelines, as their availability often depends on the healthcare system’s resources (Balea and Glasdam, 2024; Gu et al., 2022). Even when available, guidelines may not always be the most appropriate solution in every clinical scenario (Pouwels et al., 2019). To address these challenges, it is essential to maintain an up-to-date knowledge base and develop robust patient-provider relationships along with person-centered communication strategies. These measures may support both healthcare professionals and patients in making qualified and informed decisions about when antibiotics are truly necessary. Ultimately, such approaches could enhance efforts to prevent AMR at both individual and societal levels. Moreover, Spurling et al. (2017) warn that inconsistencies in antibiotic stewardship, reducing the effectiveness of evidence-based practices in managing AMR, risks weakening the overall efforts to maintain a clear and effective strategy against AMR, locally, nationally, and internationally. Hence, the current study calls for re-evaluation and development of healthcare policies prioritizing commercial interests and financial success over patient care, ensuring that ethical standards remain central to healthcare provision generally and specifically in relation to antibiotic treatment and AMR stewardship in primary care settings.

Finally, the current study’s method has strengths and limitations. The study’s multifaceted approach enhances the analysis and fosters a more robust, effective, and sustainable understanding of how professionals educate patients about antibiotic treatment and the prevention of AMR. The review was conducted following the PRISMA 2020 guidelines, ensuring a transparent, thorough, and accurate presentation of the methods, which supports the assessment of its quality (Garcia-Doval et al., 2017; Page et al., 2021). Additionally, the review was pre-registered on PROSPERO, providing access to the protocol and enabling a comparison between the registered elements and the final manuscript, thereby enhancing transparency (Schiavo, 2019). The construction of the search strings yielded a high volume of hits. The Boolean operator ‘NOT’ was deliberately avoided to prevent the exclusion of potentially relevant studies. As a result, filters for terms such as hospital*, quantitative stud* and dental care were not applied. Consequently, an extensive manual screening was required to identify the relevant articles. The systematic search was conducted with the support of an experienced university librarian to retrieve the most relevant and comprehensive literature aligned with the study’s aim, ensuring a systematic and transparent process. The screening process, data extraction and analysis were carried out alongside regular evaluations and discussions among all authors, further enhancing the study’s credibility. The included studies were assessed as being of moderate to high quality using the CASP qualitative study checklist, ensuring the findings’ credibility and relevance to the review’s aim. However, the chosen checklist can be criticized for not including a question regarding the studies’ underlying theoretical, ontological, and epistemological framework, which is also essential for assessing the quality of the studies (Long et al., 2020). Another limitation of the current study is that the review only included studies published in English, Scandinavian or Romanian, potentially excluding valuable perspectives presented in other languages. While the representation of 38 different countries in this study is a strength, the selected language may limit the transferability of the findings to other contexts. Furthermore, the limitations identified in the included studies, such as small sample sizes and unspecified healthcare professionals, are also considered limitations in the current literature review. A limitation is also the variation in national regulations on antibiotic use across the included studies. Different prescribing practices and antibiotic stewardship policies can affect the results, making it harder to compare findings and apply them to regions with different regulations.

## Conclusion

Focusing on primary care settings, the findings of this systematic review highlighted the complexity healthcare professionals face in educating patients about antibiotic use and AMR, with each profession—physicians, pharmacists, and nurses encountering unique challenges. These challenges went beyond the clinical levels, involving relational, social and personal levels. While many physicians focused on building trust and shared decision-making, although struggling with time constraints and patient expectations, pharmacists often played a key role in providing accessible advice. However, they were limited by commercial pressures and a lack of patient medical history, which impacted their ability to offer thorough education. Nurses, though less involved in prescribing, were essential in reinforcing antibiotic treatments. Relationally, the interactions between healthcare professionals and patients/other healthcare professionals were influenced by power dynamics, trust issues, and inconsistent communication. These factors often hindered the effectiveness of educational efforts regarding antibiotic use and AMR stewardship. Moreover, many patients felt dissatisfied when the provided consultations were rushed, or their questions remained unanswered, which could lead to confusion and possible misuse of antibiotics. In primary care, physicians, pharmacists and nurses operated within structural frameworks influenced by time pressures, heavy workloads, and commercial demands, limiting their capacity to provide detailed patient education. These demands made it difficult for them to meet their ethical responsibilities. On a personal level, they faced the challenge of acting on behalf of patients’ health while also managing the fear of losing patients or being perceived negatively if they did not prescribe antibiotics, even when they were aware it was not medically necessary.

Although often articulated as the most important act against the AMR crisis, this study demonstrated that providing adequate education on antibiotic use and AMR was not a straightforward path with simple solutions. Instead, it required acknowledging the multifaceted challenges that physicians, pharmacists and nurses faced on a daily basis. The complexity of these relational, social, and personal factors meant that there was no ‘quick fix’ through the implementation of evidence-based interventions alone. Future research and policymaking should focus on understanding these dynamics and creating environments that better support healthcare professionals in educating patients and tackling AMR. Given the limited research on nurses identified in this study, future studies should focus on the role of this professional group in antibiotic stewardship.

## Data Availability

The original contributions presented in the study are included in the article/supplementary material. Further inquiries can be directed to the corresponding author.
